# Optimized Magnesium Force Field Parameters for Biomolecular Simulations with Accurate Solvation, Ion-Binding, and Water-Exchange Properties in SPC/E, TIP3P-fb, TIP4P/2005, TIP4P-Ew, and TIP4P-D

**DOI:** 10.1021/acs.jctc.1c00791

**Published:** 2021-12-09

**Authors:** Kara K. Grotz, Nadine Schwierz

**Affiliations:** Department of Theoretical Biophysics, Max-Planck-Institute of Biophysics, Frankfurt am Main 60438, Germany

## Abstract

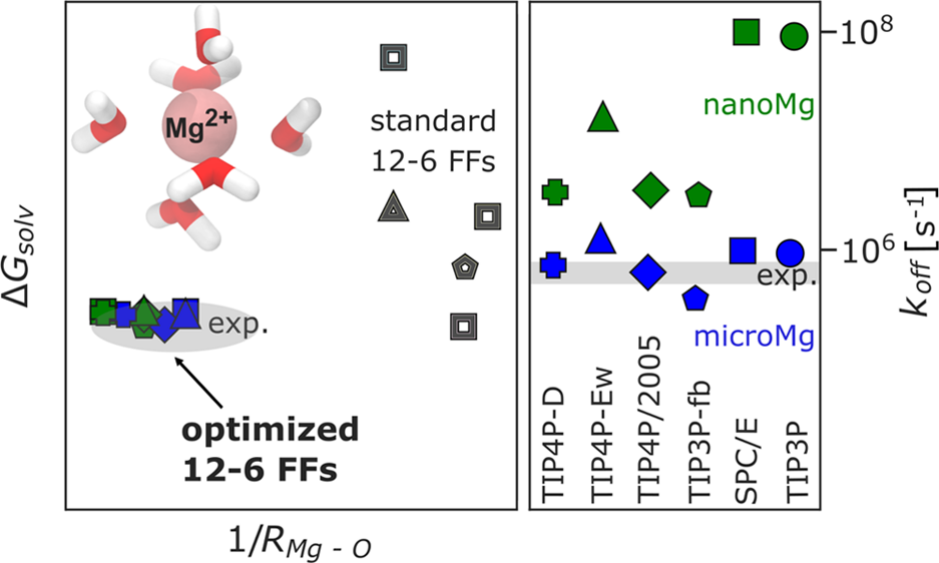

Magnesium is essential in many vital processes. To correctly describe Mg^2+^ in physiological processes by molecular dynamics simulations, accurate force fields are fundamental. Despite the importance, force fields based on the commonly used 12-6 Lennard-Jones potential showed significant shortcomings. Recently progress was made by an optimization procedure that implicitly accounts for polarizability. The resulting *microMg* and *nanoMg* force fields (*J. Chem. Theory Comput.***2021**, *17*, 2530–2540) accurately reproduce a broad range of experimental solution properties and the binding affinity to nucleic acids in TIP3P water. Since countless simulation studies rely on available water models and ion force fields, we here extend the optimization and provide Mg^2+^ parameters in combination with the SPC/E, TIP3P-fb, TIP4P/2005, TIP4P-Ew, and TIP4P-D water models. For each water model, the Mg^2+^ force fields reproduce the solvation free energy, the distance to oxygens in the first hydration shell, the hydration number, the activity coefficient derivative in MgCl_2_ solutions, and the binding affinity and distance to the phosphate oxygens on nucleic acids. We present two parameter sets: *MicroMg* yields water exchange on the microsecond time scale and matches the experimental exchange rate. Depending on the water model, *nanoMg* yields accelerated water exchange in the range of 10^6^ to 10^8^ exchanges per second. The *nanoMg* parameters can be used to enhance the sampling of binding events, to obtain converged distributions of Mg^2+^, or to predict ion binding sites in biomolecular simulations. The parameter files are freely available at https://github.com/bio-phys/optimizedMgFFs.

## 1 Introduction

Magnesium is the second most abundant intracellular cation.^1^ It is involved in more than 600 enzymatic reactions^2^ and plays a crucial role in vital processes such as ATP hydrolysis,^3^ cellular signaling,^2^ or the catalytic activity of ribozymes.^4^ The specific requirement for Mg^2+^ is prevalent in nucleic acid systems where Mg^2+^ is crucial for the stability, folding, and biological function.^5−9^ In addition to its physiological relevance, Mg^2+^ is important in DNA nanotechnology^10−12^ and is a promising candidate for divalent batteries.^13,14^

Due to the distinct role of Mg^2+^, the modeling of Mg^2+^ by all-atom molecular dynamics (MD) simulations has received significant attention.^15−26^ Despite tremendous efforts, Mg^2+^ force fields based on the commonly used 12-6 Lennard-Jones interaction potential failed to provide a quantitative description (see Table S1) for three reasons. (i) No parameter combination existed that simultaneously reproduced the solvation free energy and the size of the first hydration shell.^20,21,23,27^ (ii) The parameters yielded too low water exchange rates leading to unrealistically slow exchange kinetics such that transitions from outer to inner sphere binding and back could never be observed on the typical time scale of MD simulations.^28^ (iii) The binding affinity of Mg^2+^ to ion binding sites on biomolecules was overrated significantly.^19,25,29,30^ Regarding these shortcomings, the immediate question arises why classical, nonpolarizable Mg^2+^ force fields fail to provide an accurate description. Clearly, Mg^2+^ ions strongly polarize their environment, and the lack of charge transfer and polarization effects in classical simulations likely leads to the observed deviations between experiments and simulations. Possibilities to provide improvement include the use of computationally more demanding polarizable force fields,^31^ to include additional parameters in the interaction potential,^22,30,32^ or to scale the charge of the Mg^2+^ ion.^18,33^ Another possibility is to include the effect of polarizability implicitly without introducing additional terms in the interaction potential. As shown in our previous work,^26^ polarizability can be taken into account by two measures. First, optimizing the parameters of the 12-6 Lennard-Jones parameters in an enlarged parameter space captures the more attractive and long-ranged Mg^2+^–water interactions, thereby facilitating the simultaneous matching of the experimental solvation free energy, size of the first hydration shell, and water exchange rate. Second, by introducing scaling factors^34,35^ in the standard combination rules for the Mg^2+^–Cl^–^ and Mg^2+^–RNA interactions, deviations due to polarization are taken into account, and the experimental activity derivative and the binding affinity to the phosphate oxygen on nucleic acids is reproduced. The resulting force field parameters provide an efficient and highly accurate model for Mg^2+^ in biomolecular simulations in combination with the TIP3P water.

The TIP3P water model is frequently used in biomolecular simulations since protein and nucleic acid force fields were frequently optimized in the presence of TIP3P. However, to date, a large variety of different water models exist, some of which reproduce the physical properties of water better compared to TIP3P.^36^ In principle, it is possible to combine more advanced water models and ion parameters with the force fields for biomolecules. In some cases, such a combination can leverage the strengths of the respective parameters and improve the agreement between simulated and experimental structures.^25,37−42^ However, the combination of force field parameters does not guarantee that the physical properties, which were targeted in the first place, are reproduced. For metal cations, previous studies indicate limited transferability and different water models can have significant effects on the solvation free energy, the exchange kinetics, and even the reaction mechanism.^43−45^ It is therefore crucial to assess the transferability of Mg^2+^ parameters to different water models and optimize the force field parameters if necessary in order to ensure physically meaningful results.

The aim of our current work is to provide optimized Mg^2+^ force field parameters in combination with five different water models. We focus on some of the most popular rigid nonpolarizable water models, namely, SPC/E,^46^ TIP3P-fb,^47^ TIP4P/2005,^48^ TIP4P-Ew,^49^ and TIP4P-D.^50^ Our results illustrate that the transferability of the Mg^2+^ parameters^26^ developed in combination with TIP3P is limited. In particular, for the 4-site water models, significant deviations from the experimental properties are observed, in agreement with similar studies.^44^ In order to provide accurate parameters for the different water models, we systematically derive Mg^2+^ parameters that reproduce the solvation free energy, the distance to oxygens in the first hydration shell, the hydration number, the activity coefficient derivative in MgCl_2_ solutions, and the binding affinity and distance to nonbridging phosphate oxygens on nucleic acids, using our previously developed optimization procedure.^26^ In order to provide consistent and robust results, Cl^–^ was chosen as the reference ion, and its parameters were optimized in a preceding step. For each water model, we present two parameter sets: *MicroMg* yields water exchange on the microsecond time scale and matches the experimental exchange rate. *NanoMg* yields accelerated water exchange in the range of 10^6^ to 10^8^ exchanges per second depending on the water model used. The *nanoMg* parameters are suited to accelerate the binding kinetics in biomolecular simulations and improve the sampling of ionic distributions. Our results reveal that the largest speed-up is obtained in combination with TIP3P or SPC/E.

## 2 Methods

### 2.1 Molecular Dynamics Simulations

The ions are modeled as point charges, and the electrostatic, dispersion, and excluded volume interactions are taken into account by a pairwise interaction potential. We use the most common form of the Lennard-Jones (LJ) potential with a repulsive *r*^–12^ and an attractive *r*^–6^ term
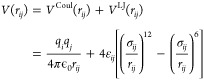
1where *q*_*i*_ and *q*_*j*_ are the charges of atoms *i* and *j*, *r*_*ij*_ is the distance between them, and ϵ_0_ is the dielectric constant of the vacuum. The parameters σ_*ij*_ and ε_*ij*_ describe the LJ diameter and interaction strength, respectively. Following the procedure to optimize the force field parameters for Mg^2+^ in TIP3P water from our previous work,^26^ we systematically optimize the two parameters of the LJ potential and the combination rules for 5 different nonpolarizable rigid water models. From the different three site models, we chose the most commonly used SPC/E^46^ and the more recent TIP3P-fb^47^ water model. From the different four site models, we chose the TIP4P/2005,^48^ TIP4P-Ew,^49^ and TIP4P-D.^50^ TIP4P/2005 has gained popularity and is often quoted as the best nonpolarizable general-purpose model.^36^ In addition to TIP3P, TIP4P-Ew is frequently used in the AMBER and CHARMM force fields.^30,51,52^ TIP4P-D is one of the newer offsprings in the TIP4P family and was designed to improve water dispersion interactions.

Partial charges, Lennard-Jones parameters, bond lengths, and bond angles of the different waters are shown in Section S1.2.

Since the standard combination rules do not take polarizability and charge transfer effects into account, we use scaling parameters in the Lorentz–Berthelot combination rules to describe the Mg^2+^–Cl^–^ and the Mg^2+^–RNA interactions.^26^ Following the work by Fyta and Netz^35^ and previous similar studies,^29,34,53^ the modified Lorentz–Berthelot combination rules have the following from
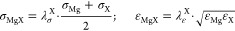
2where X denotes Cl^–^ or the atoms of the RNA. Note that the solvation free energy, the structural properties of the first hydration shell, and the rate of water exchange remain unchanged upon changing the Mg^2+^–Cl^–^ and the Mg^2+^–RNA combination rule. In this work four scaling parameters are introduced, λ_σ_^RNA^, λ_ϵ_^RNA^, λ_σ_^Cl^, and λ_ϵ_^Cl^, to optimize ion–ion interactions and Mg^2+^–DMP interactions. Note that, in the RNA system, the same scaling factors λ_σ_^RNA^ and λ_ϵ_^RNA^ are applied to all atoms of the RNA.

To compare the optimized parameters (Table 1) to the available force fields from the literature, we performed simulations using the 12-6 based parameters by Åquist,^15^ Mamatkulov–Netz,^20^ and Li–Merz^21^ (HFE set) and the 12-6-4 based parameters by Li–-Merz^22^ for SPC/E water. In addition, the parameters by Li–Merz^21,22,32^ (12-6 and 12-6-4) for TIP4P-Ew and TIP3P-fb were used (data not shown). For SPC/E water, the Cl^–^ parameters were taken from Smith–Dang.^54^ For all other water models, the Cl^–^ parameters were adjusted as described below.

**Table 1:** Optimized Force Field Parameters for Mg^2+^ for Simulations with Different Water Models^a^

	σ_ii_ [nm]	ε_ii_ [kJ/mol]	σ_io_ [nm]	ε_io_ [kJ/mol]	λ_σ_^Cl^	λ_ε_^Cl^	σ_MgCl_ [nm]	ε_MgCl_ [kJ/mol]	λ_σ_^RNA^	λ_ε_^RNA^	σ_MgOP_ [nm]	ε_MgOP_ [kJ/mol]
TIP3P												
*microMg*(26)	0.1019	235.80	0.2085	12.25	1.80	0.1	0.4878	0.8181	1.1375	0.3200	0.2262	4.6061
*nanoMg*(26)	0.1025	389.80	0.2088	15.75	1.80	0.1	0.4884	1.0518	1.1435	0.2500	0.2277	4.6266
SPC/E												
*microMg*	0.1036	290.58	0.2101	13.75	1.59	0.1	0.4318	1.0989	1.1019	0.4856	0.2202	7.7589
*nanoMg*	0.1046	470.70	0.2106	17.50	1.59	0.1	0.4325	1.3986	1.1107	0.3300	0.2225	6.7111
TIP3P-fb												
*microMg*	0.1032	311.38	0.2105	14.25	1.59	0.1	0.4743	0.3983	1.0957	0.4913	0.2187	8.1266
*nanoMg*	0.1034	380.38	0.2106	15.75	1.59	0.1	0.4744	0.4403	1.1002	0.4172	0.2197	7.6266
TIP4P/2005												
*microMg*	0.0901	712.67	0.2030	23.50	1.59	0.1	0.4719	0.5529	1.1484	0.2648	0.2217	6.6266
*nanoMg*	0.0913	774.62	0.2036	24.50	1.59	0.1	0.4728	0.5764	1.1345	0.2923	0.2217	6.6266
TIP4P-Ew												
*microMg*	0.0910	647.63	0.2037	21.00	1.59	0.1	0.4684	0.5622	1.1456	0.2778	0.2217	6.6266
*nanoMg*	0.0926	760.06	0.2045	22.75	1.59	0.1	0.4697	0.6096	1.1489	0.2371	0.2232	6.1266
TIP4P-D												
*microMg*	0.0960	621.50	0.2057	20.08	1.59	0.1	0.4811	0.4697	1.3255	0.3264	0.2197	7.6266
*nanoMg*	0.0970	680.01	0.2062	21.00	1.59	0.1	0.4819	0.4913	1.1231	0.2834	0.2207	6.9266

a σ_ii_, ε_ii_, σ_io_, and ε_io_ are the ion–ion and ion–water LJ parameters. λ_σ_^X^ and λ_ε_^X^ are the scaling factors for the Lorentz–Berthelot combination rules (eq 2) for the interaction with Cl^–^ or the RNA atoms, shown exemplary for the interaction between Mg^2+^ and OP. Note that the scaling factors are only valid in combination with the Cl^–^ parameters from Smith–Dang^54^ for SPC/E water and those listed in Table 2 for the other water models, and the parmBSC0χ_OL3_ RNA parameters.^84−86^ Values for parameters in TIP3P are taken from ref (26).

Simulations with force fields of the 12-6 type were performed with GROMACS^55^ (versions 2018, 2020). Simulations with force fields of the 12-6-4 type were done with AMBER^52^ (version 2018) and PLUMED^56^ (version 2.6.4) since GROMACS does not support 12-6-4 interaction potentials. Section S1.3 (Tables S3 and S4) lists all simulation setups. Similar to previous work,^19,26,30,57,58^ we used dimethylphosphate (DMP) to optimize the ion–RNA interactions. The force field parameters for the DMP molecule are based on a parametrization with GAFF^59^ and can be found in ref (26). The analysis was performed with built-in GROMACS^55^ code and by using the MDAnalysis package^60,61^ for python.

### 2.2 Optimization Procedure

The optimization procedure follows the same three step strategy described in ref (26), starting with an optimization of the ion–water interaction by performing a grid search in σ_io_–ε_io_ space. Here, in a first step, all σ_io_–ε_io_ parameter combinations matching the experimental solvation free energy Δ*G*_solv_, the Mg^2+^-oxygen distance in the first hydration shell *R*_1_, and the coordination number *n*_1_ are selected.

Second, by calculating the rate of water exchange for the above-mentioned parameter combinations, we optimize the water exchange dynamics. For each water model two parameter sets were chosen: The *microMg* parameter sets yield water exchange on the microsecond time scale and reproduce the experimental rate within errors. The second parameter sets yield faster water exchange time scales providing exchange dynamics that range between 10^6^ to 10^8^ exchanges per second while simultaneously reproducing all thermodynamic and structural properties. For consistency with our previous work,^26^ we refer to the second set as *nanoMg*, even though the nanosecond time scale could not be reached for all water models.

In the final step, we optimize the ion–ion and ion–RNA interactions by calculating activity coefficient derivatives and ion binding affinities. We performed a grid search in λ_σ_^X^ and λ_ε_^X^ parameter space (eq 2). The activity coefficient derivatives *a*_cc_ are collected and the scaling factors λ_σ_^Cl^ and λ_ε_^Cl^ are selected by reproducing the experimental activity derivative over a broad range of MgCl_2_ concentrations, using the Kirkwood–Buff theory.^62^ To calculate the binding affinity of Mg^2+^ toward one of the nonbridging phosphate oxygens of DMP, we used alchemical transformation calculations. Reproducing the experimental binding affinity Δ*G*_b_^0^ and binding distance *R*_b_ toward the phosphate oxygen, we select the scaling factors λ_σ_^RNA^, λ_ε_^RNA^.

### 2.3 Solvation Free Energy

The solvation free energy is considered the most important thermodynamic property in the development of accurate force field parameters.^27^ Yet, the proton solvation free energy can vary depending on the experimental sources. In most cases, the solvation free energy of the proton by Tissandier et al.,^63^ −1104 kJ/mol, or Marcus,^64^ −1056 kJ/mol, are used. Since, depending on the exact choice, the estimates can vary by more than 50 kJ/mol, we take the more robust solvation free energy of neutral ion pairs into account. In the following, we use chloride as the reference ion as in previous works.^20,23,27^ In order to obtain consistent results, appropriate corrections for finite size effects, compression, and interfacial crossing have to be applied. Finite size effects are taken into account via^65^
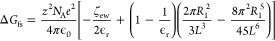
3where *z* is the valency, *N*_A_ Avogardo’s number, *e* the unit charge, ϵ_0_ the vacuum permittivity, and *R*_1_ the first peak of the ion–water radial distribution function. Hence, the effective ion radius, ζ_ew_ = −2.837 279/*L*, is the Wigner potential, with *L* being the edge length of the simulation box in nanometers.^65,66^ ϵ_r_ is the relative dielectric constant of the different water models. We used ϵ_r_(SPC/E) = 68, ϵ_r_(TIP3P-fb) = 81.3, ϵ_r_(TIP4P/2005) = 58, ϵ_r_(TIP4P-Ew) = 63, and ϵ_r_(TIP4P-D) = 68. The values for SPC/E and TIP4P/2005 are taken from ref (36), and all other values are from their original publications.^47,49,50^

The correction term related to the compression of the gas is given by^27^
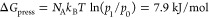
4and is independent from the choice of the water model, with *p*_0_ = 1 atm being the pressure of ideal gas and *p*_1_ = 24.6 atm corresponding to an ideal solution under pressure at a density of 1 mol/L. In the experiments,^63^ the ions have to pass the air–water interface in order to enter into the aqueous phase. The corresponding free energy correction term is^27^
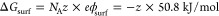
5Here, we chose the surface potential as ϕ_surf_ = −0.527 V obtained for polarizable TIP4P-FQ water.^67,68^ The reason is that this choice closely matches with the experimentally based estimation of −0.50 V.^69^ Even more importantly, the interfacial crossing term almost exactly cancels the shift between the solvation free energies by Marcus^64^ and by Tissandier^63^ for different anions and cations. Consequently, we obtain consistent results if we compare the simulation results with the interfacial crossing term to the free energies by Tissandier (since those values include a full contribution from the bulk water surface potential^69^) or if we compare the simulation results without interfacial crossing term to the bulk free energies by Marcus (which omit the surface potential^69^).

Using the former approach, the solvation free energy is given by
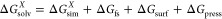
6The solvation free energy of the neutral MgCl_2_ ion pairs is given by
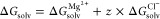
7

### 2.4 Parametrization of Cl^–^ and Mg^2+^

**Figure 1:**
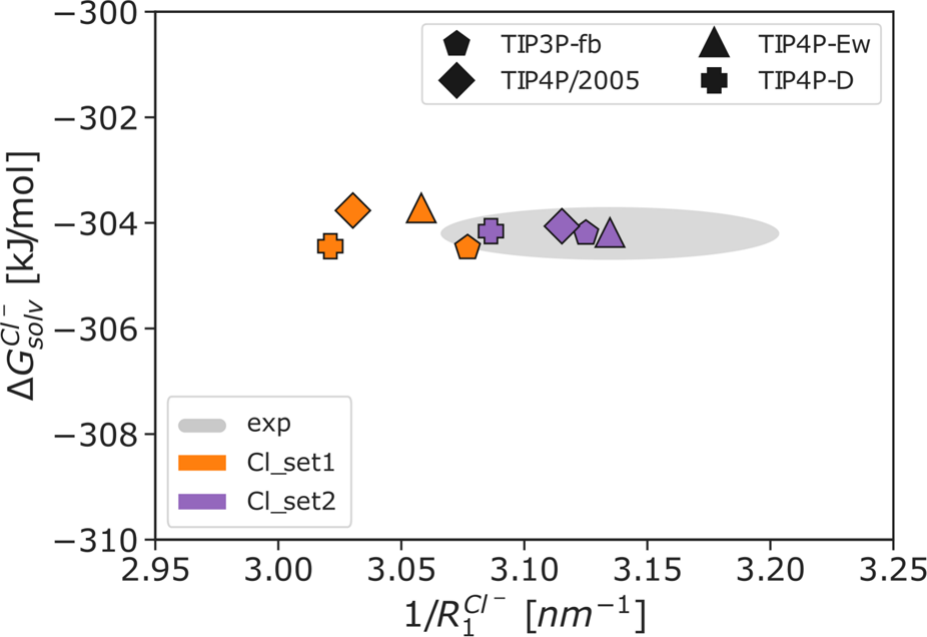
Solvation free energy Δ*G*_solv_^Cl–^ of Cl^–^ in correlation with the inverse of the Cl^–^–oxygen distance of the first hydration shell 1/*R*_1_^Cl–^. The gray area indicates the experimental results from refs (64 and 87). One set of parameters is obtained by starting from Smith–Dang parameters^54^ for SPC/E water and modifying the LJ parameter ε_io_ (orange, Cl_set1). The second set is obtained by starting from Joung–Cheatham parameters^70^ for TIP4P-Ew water and modifying the LJ parameter σ_io_ (purple, Cl_set2).

Initially, we optimize the parameters of the reference chloride ion. For SPC/E water, we use the well established Smith–Dang parameters.^54^ In particular, the calculated solvation free energy is Δ*G*_solv_^Cl–^ = −306 kJ/mol^27^ and closely matches the experimental results by Tissandier^63^ (−304.2 kJ/mol). For the optimization of the parameters for Cl^–^ in combination with TIP3P-fb, TIP4P/2005, TIP4P-Ew, and TIP4P-D water, we used two different approaches: In the first approach, we modified ε_io_ starting from the Smith–Dang parameters^54^ (σ_io_ = 0.378 nm, ε_io_ = 0.52 kJ/mol). We selected the values for ε_io_ that reproduced the experimental results while keeping σ_io_ fixed. In the second approach, we modified σ_io_ starting from the Joung–Cheatham chloride parameters^70^ for TIP4P-Ew water (σ_io_ = 0.404 105 546 nm, ε_io_ = 0.182 336 936 kJ/mol). We selected the values for σ_io_ that reproduced the experimental results while keeping ε_io_ fixed. As shown in Figure 1, the second approach allowed us to simultaneously reproduce Δ*G*_solv_^Cl–^ and *R*_1_, the radius of the first hydration shell, for all water models. The parameters from the second approach were therefore used in the optimization of Mg^2+^. The resulting LJ parameters are listed in Table 2.

**Table 2:** Optimized Force Field Parameters for Cl^–^ for Simulations with Different Water Models^a^

	σ_ii_ [nm]	ε_ii_ [kJ/mol]	σ_io_ [nm]	ε_io_ [kJ/mol]	Δ*G*_solv_^Cl–^ [kJ/mol]	*R*_1_^Cl–^ [nm]
Cl(TIP3P-fb)	0.493 358	0.050 960	0.4056	0.1823	–304.2 ± 1	0.320
Cl(TIP4P/2005)	0.503 464	0.042 887	0.4097	0.1823	–304.1 ± 1	0.321
Cl(TIP4P-Ew)	0.498 225	0.048 805	0.4073	0.182 3	–304.2 ± 1	0.324
Cl(TIP4P-D)	0.509 112	0.035 496	0.4128	0.1823	–304.2 ± 1	0.319
exp.					–304.2^63^	0.319 ± 0.007^87^

a σ_ii_ and ε_ii_ are the ion–ion and σ_io_ and ε_io_ the ion–water LJ parameters. The parameters were obtained starting from Joung–Cheatham parameters^70^ for TIP4P-Ew and modifying the LJ parameter σ_io_ until the experimental single-ion properties Δ*G*_solv_^Cl–^ and *R*_1_^Cl–^ are matched. The experimental value for *R*_1_^Cl–^ uses Tissandier’s estimate for the proton solvation free energy.^63^

Subsequently, the solvation free energy for Mg^2+^ was calculated on a grid with σ_io_ = 0.195–0.22 nm and ε_io_ = 0.02–28 kJ/mol. In addition, *R*_1_, *n*_1_, and the self-diffusion coefficient *D*_0_ were calculated for all waters as described in ref (26).

### 2.5 Free Energy Profiles, Water Exchange Rate, Activity Derivative, and Binding Affinity

We used umbrella sampling^71,72^ to calculate one- and two-dimensional free energy profiles, straightforward simulations to calculate the water exchange rate (see Section S1.5 for more details), Kirkwood–Buff theory^62^ to calculate the activity derivative with respect to the natural logarithm of the number density of the Mg^2+^ ions (see Section S1.6 for more details), and alchemical transformation to calculate the binding affinities. Additional details regarding the calculation of free energy profiles (Section S1.7) and binding affinities (Section S1.8) can be found in the Supporting Information of this work.

## 3 Results and Discussion

In the following, we present the results from our parameter optimization of Mg^2+^ in combination with the SPC/E, TIP3P-fb, TIP4P/2005, TIP4P-Ew, and TIP4P-D water models. Our optimization procedure is done in three sequential steps^26^ and allows us to reproduce a broad range of thermodynamic properties including the solvation free energy, the distance to oxygens in the first hydration shell, the hydration number, the activity coefficient derivative in MgCl_2_ solutions, and the binding affinity and distance to the phosphate oxygens of RNA (Tables 3 and 5). For each water model, we present two sets of optimal parameters: *MicroMg* yields water exchange on the microsecond time scale and matches the experimental exchange rate. *NanoMg* yields accelerated water exchange in the range of 10^6^ to 10^8^ exchanges per second depending on the water model used (Table 4).

### 3.1 Transferability of Ion Parameters between Different Water Models

**Figure 2:**
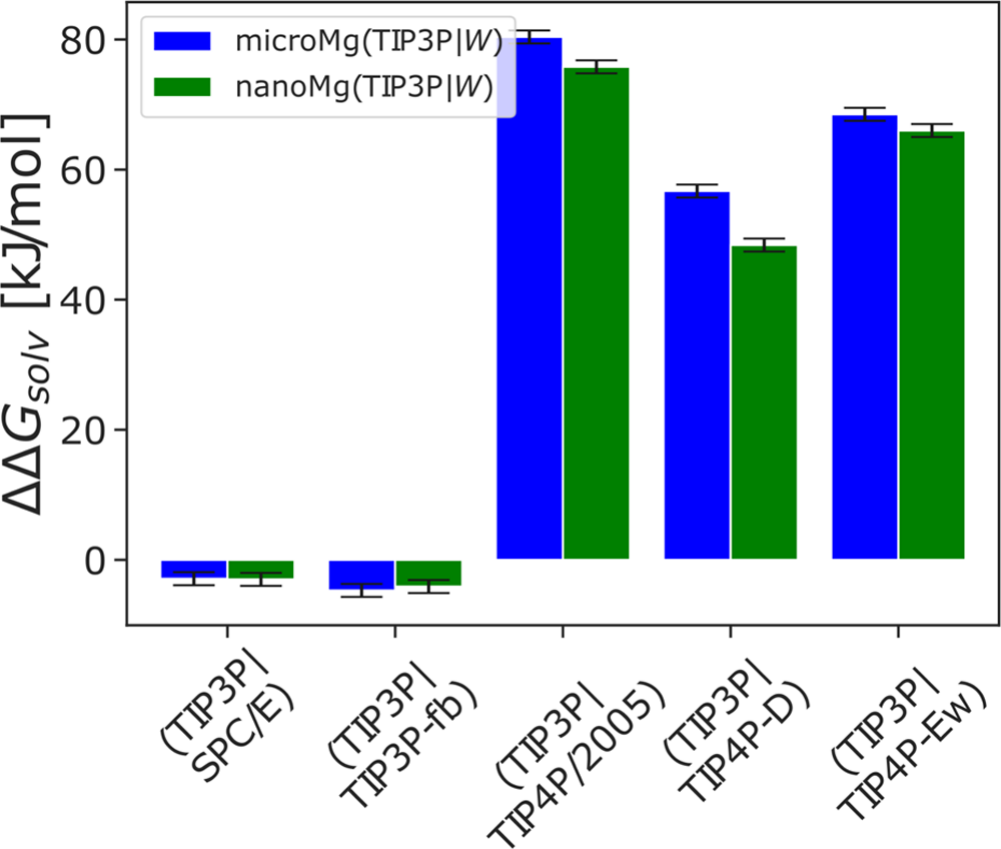
Difference in solvation free energy to the experimental value of −2532 kJ/mol^64^ for parameters transferred to different water models. The *microMg* and *nanoMg* parameters^26^ that have been optimized in TIP3P are used with one the following water models *W* (SPC/E,^46^ TIP3P-fb,^47^ TIP4P/2005,^48^ TIP4P-Ew,^49^ and TIP4P-D^50^), resulting in the converted parameter sets *microMg*(TIP3P|*W*) and *nanoMg*(TIP3P|*W*). Additional single-ion properties are shown in Table S7.

We tested the transferability of the recently developed Mg^2+^ parameters^26^*microMg*(TIP3P) and *nanoMg*(TIP3P), that were optimized in combination with the TIP3P water model. Figure 2 and Table S3 show that the transferability of the parameters to other water models is limited and deviations from the experimental solvation free energy are observed. While for the 3-site water models (TIP3P-fb and SPC/E) the deviations are small (3–4 kJ/mol), significant deviations up to 80 kJ/mol are observed for the 4-site models (TIP4P/2005, TIP4P-Ew, and TIP4P-D). The influence of the different water models on the solvation free energy Δ*G*_solv_, the radius *R*_1_ of the first hydration shell, and the coordination number *n*_1_ is also reflected in the free energy isolines which display differences between 3- and 4-site water models (Figure S3). Since the transferability of the Mg^2+^ parameters is limited, in agreement with a similar previous study on metal cations,^44^ we have systematically optimized the parameters in combination with 5 different water models.

### 3.2 Optimization of Solvation Free Energy, Radius of Hydration Shell, and Coordination Number

**Figure 3:**
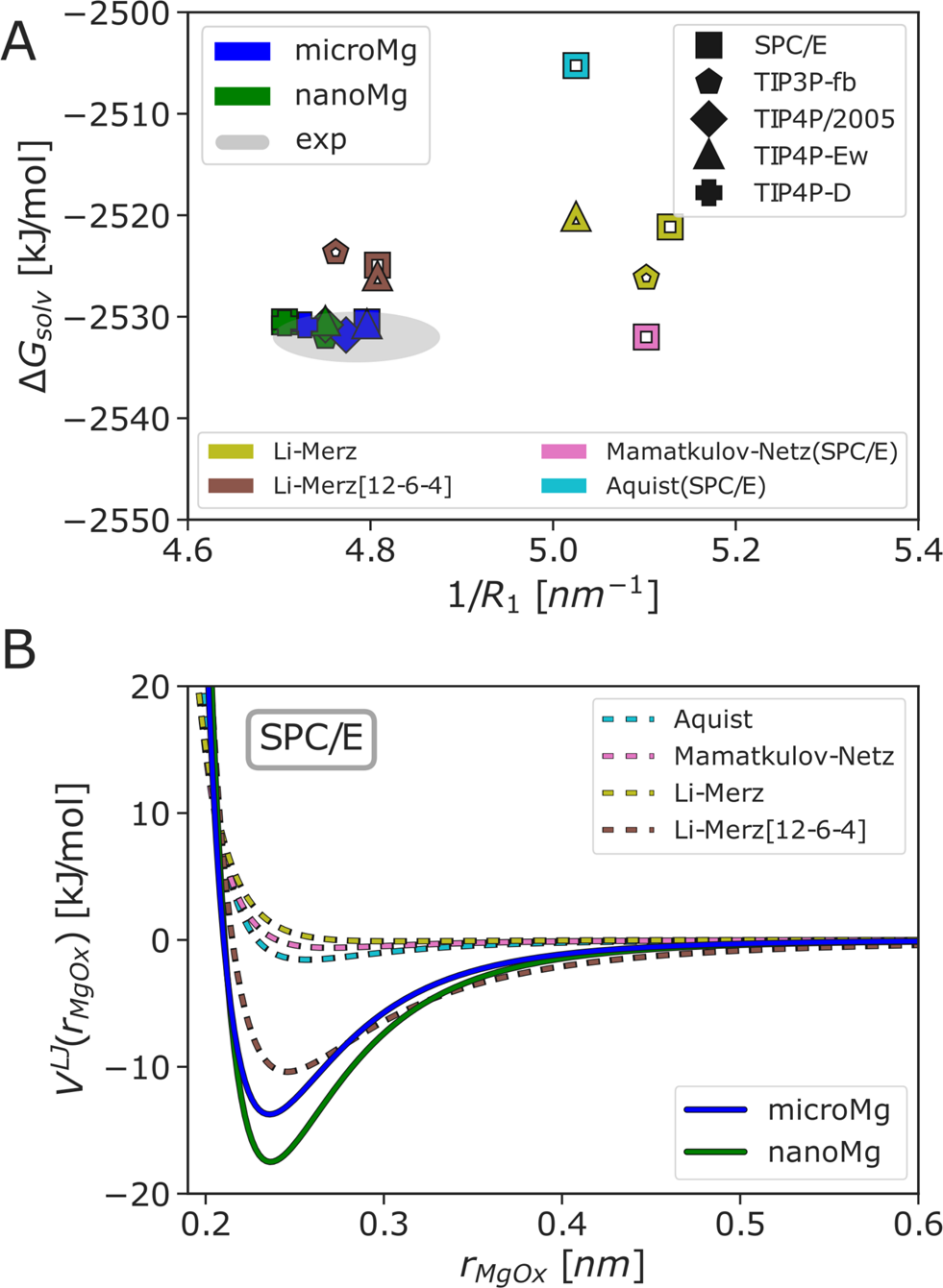
Comparison of the optimized parameter sets *microMg* (blue) and *nanoMg* (green) of various water models (SPC/E,^46^ TIP3P-fb,^47^ TIP4P/2005,^48^ TIP4P-Ew,^49^ and TIP4P-D^50^) with force fields from the literature^15,20−22,32^ and experimental data. (A) Solvation free energy Δ*G*_solv_ for neutral MgCl_2_ pairs in correlation with the inverse of the Mg^2+^–oxygen distance of the first hydration shell 1/*R*_1_. The gray area indicates the experimental results from refs (64 and 87). The different marker shapes indicate Mg^2+^ models of different water models. The colors indicate the different Mg^2+^ models. The values for Åquist were taken from ref (89). Solvation free energy values for parameters by Li–Merz (both 12-6 and 12-6-4 based) and Åquist were combined with Cl^–^ values by Marcus.^87^ (B) Lennard-Jones interaction potential *V*^LJ^ as a function of the Mg^2+^–oxygen distance *r*_MgOx_ for different Mg^2+^ force fields and exemplary SPC/E water. Similar plots for the *microMg* and *nanoMg* parameters of the other water models can be found in Figure S4.

For all 5 water models, we selected parameter combinations for σ_io_ and ε_io_ that accurately reproduce Δ*G*_solv_, *R*_1_, and *n*_1_ simultaneously (Figure 3A and Table 3). Hereby, the key to a successful parametrization was to take polarizability into account implicitly by considering a larger range of possible LJ parameters.^26^ As shown exemplary for SPC/E water in Figure 3B (for the other waters see Figure S4), the interaction potentials with our optimized parameter sets *microMg* and *nanoMg* are more long-ranged compared to the standard parameter sets from the literature^15,20,21^ and similar to 12-6-4 potentials, which explicitly consider ion–dipole interactions via additional terms in the interaction potential.^22^ Thus, our optimized parameters take polarization effects into account implicitly, and the interaction between Mg^2+^ and water is more attractive and long-ranged compared to the standard force fields from the literature. This in turn allows us to reproduce the experimental results for Δ*G*_solv_, *R*_1_, and *n*_1_ simultaneously and to achieve agreement comparable to 12-6-4 interaction potentials^22^ (Figure 3A) without introducing additional parameters in the functional form of the interaction potential (eq 1).

**Table 3:** Results for Single-Ion and Ion–Ion Properties for the Optimized Parameters in Direct Comparison with Experimental Results^a^

	Δ*G*_solv_ [kJ/mol]	*R*_1_ [nm]	*n*_1_	*D*_0_ [10^–5^ cm^2^/s]	*a*_cc_
*microMg*(TIP3P)^26^	–2532.9 ± 1	0.207 ± 0.004	6	0.754 ± 0.006	0.93 ± 0.01
*nanoMg*(TIP3P)^26^	–2532.0 ± 1	0.209 ± 0.004	6	0.750 ± 0.004	0.97 ± 0.01
*microMg*(SPC/E)	–2530.5 ± 1	0.209 ± 0.004	6	0.475 ± 0.004	0.76 ± 0.02
*nanoMg*(SPC/E)	–2530.5 ± 1	0.213 ± 0.004	6	0.497 ± 0.009	0.86 ± 0.03
*microMg*(TIP3P-fb)	–2531.3 ± 1	0.211 ± 0.004	6	0.509 ± 0.014	0.96 ± 0.05
*nanoMg*(TIP3P-fb)	–2532.0 ± 1	0.211 ± 0.004	6	0.463 ± 0.017	0.95 ± 0.02
*microMg*(TIP4P/2005)	–2531.8 ± 1	0.210 ± 0.004	6	0.548 ± 0.007	0.87 ± 0.04
*nanoMg*(TIP4P/2005)	–2530.8 ± 1	0.211 ± 0.004	6	0.495 ± 0.013	0.84 ± 0.05
*microMg*(TIP4P-Ew)	–2530.9 ± 1	0.209 ± 0.004	6	0.475 ± 0.011	0.85 ± 0.03
*nanoMg*(TIP4P-Ew)	–2530.8 ± 1	0.211 ± 0.004	6	0.469 ± 0.008	0.87 ± 0.05
*microMg*(TIP4P-D)	–2530.8 ± 1	0.212 ± 0.004	6	0.531 ± 0.016	1.01 ± 0.02
*nanoMg*(TIP4P-D)	–2530.6 ± 1	0.213 ± 0.004	6	0.530 ± 0.013	0.89 ± 0.03
exp.	–2532^64^	0.209 ± 0.004^87^	6^87^	0.706^64^	0.93^88^

a Solvation free energy of neutral MgCl_2_ ion pairs Δ*G*_solv_, Mg^2+^–oxygen distance in the first hydration shell *R*_1_, coordination number of the first hydration shell *n*_1_, self-diffusion coefficient *D*_0_, and *a*_cc_ the activity derivative of a MgCl_2_ solution at 0.25 M concentration. Values for parameters in TIP3P are taken from ref (26).

### 3.3 Optimization of the Water Exchange Rate

**Figure 4:**
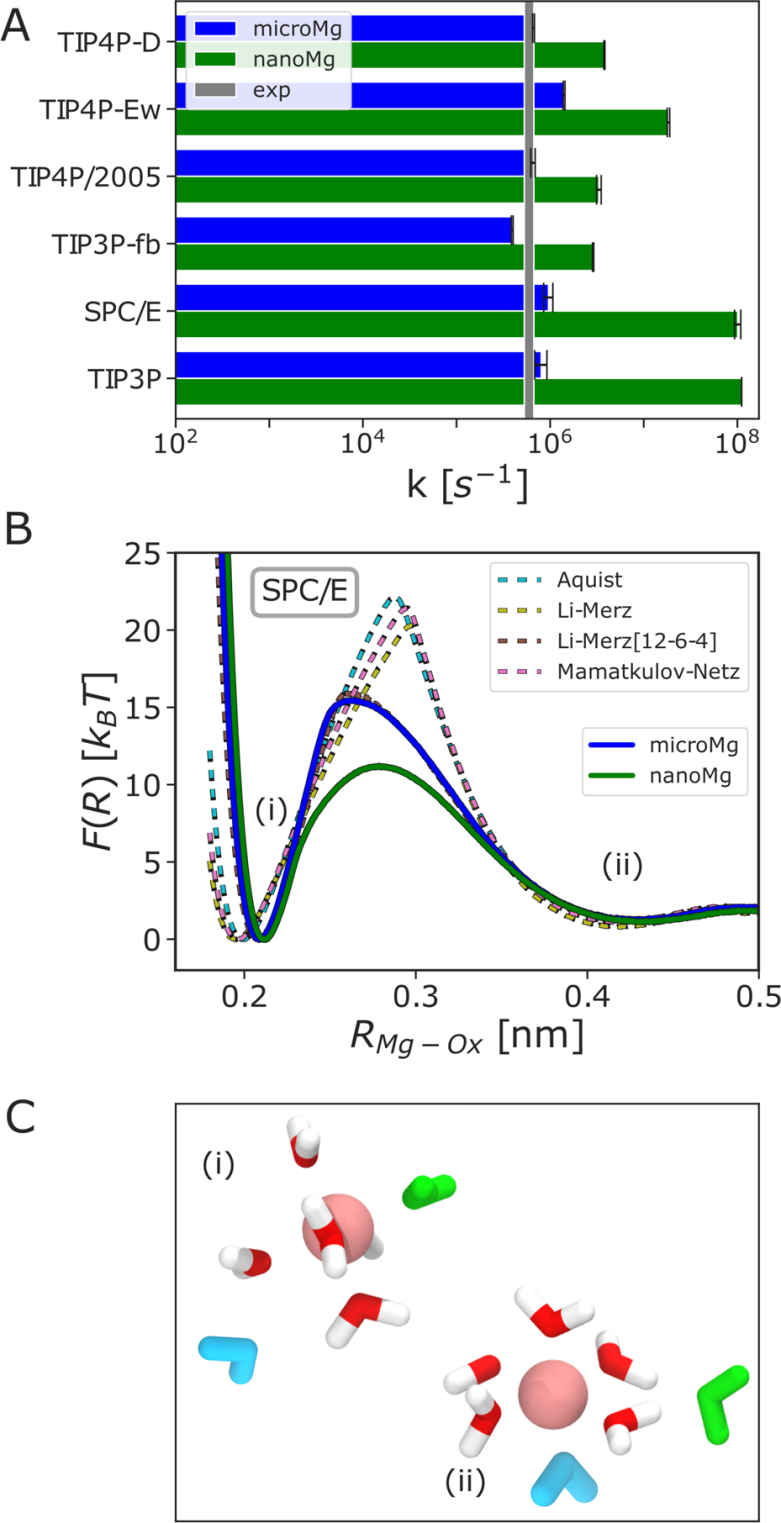
Water exchange in the first hydration shell of Mg^2+^. (A) Kinetic rate coefficients *k* (eq S3) of *microMg* (blue) and *nanoMg* (green) of different water models (SPC/E,^46^ TIP3P-fb,^47^ TIP4P/2005,^48^ TIP4P-Ew,^49^ and TIP4P-D^50^) and for TIP3P from the literature.^26^ The gray vertical line indicates the experimental values^73,74^ (Table 4). (B) One-dimensional free energy profiles as a function of the distance between Mg^2+^ and the leaving water molecule *R*_Mg–Ox_ for different force fields in combination with SPC/E water. (C) The snapshots show representative conformations in the two stable states: (i) Before exchange: Leaving water (shown in green) is part of the first and entering water (shown in blue) is part of the second hydration shell. (ii) After exchange: Leaving water is in the second hydration shell and the entering water molecule filled the void in the first hydration shell. The snapshots were taken using *microMg*(SPC/E).

In the next step of our optimization, we selected two sets of parameters from the data that reproduced Δ*G*_solv_, *R*_1_, and *n*_1_ in the previous step based on the calculated water exchange rate. In order to provide an accurate estimate of the rate constant, the rate was calculated from 1 μs long straightforward simulations since transition state theory, albeit computationally less demanding, can overestimate the true rate by more than 2 orders of magnitude.^28^ The rates for the two sets of parameters for all water models are shown in Figure 4A and Table 4. The first parameter sets, *microMg*, yield water exchange on the microsecond time scale in agreement with experimental results.^73,74^ The second parameter sets, *nanoMg*, yield accelerated water exchange with 10^6^ to 10^8^ exchanges per second dependent on the water model used (Table 4). In all cases, the parameters for *nanoMg* were chosen such that they yield the highest rate while still reproducing all other experimental properties. Yet, the different properties of the water models,^36^ in particular the dielectric constant of the models, lead to distinct differences in the parameter range that reproduces Δ*G*_solv_, *R*_1_, and *n*_1_ (Figure S3). Consequently, the maximum exchange rate that could be achieved differs for the different water models. *NanoMg*(SPC/E) yields the highest exchange rate with 10^8^ exchanges per second, similar to the acceleration achieved for TIP3P.^26^*NanoMg*(TIP4P-Ew) yields the next highest rate with 10^7^ s^–1^ followed by *nanoMg*(TIP3P-fb), *nanoMg*(TIP4P/2005), and *nanoMg*(TIP4P-D) with 10^6^ exchanges per second (Table 4 and Figure 4A).

**Table 4:** Properties of Water Exchange from Simulations and Experiments^a^

	*N*	*k* [s^–1^]	ΔF* [k_B_T]
*microMg*(TIP3P)^26^	376 ± 56	(8.04 ± 1) × 10^5^	15.9
*nanoMg*(TIP3P)^26^	52 086 ± 120	(1.11 ± 0.003) × 10^8^	11.5
*microMg*(SPC/E)	452 ± 52	(9.62 ± 1) × 10^5^	15.5
*nanoMg*(SPC/E)	47 472 ± 3620	(1.01 ± 0.08) × 10^8^	11.2
*microMg*(TIP3P-fb)	184 ± 4	(3.94 ± 0.09) × 10^5^	16.1
*nanoMg*(TIP3P-fb)	1344 ± 20	(2.88 ± 0.05) × 10^6^	14.1
*microMg*(TIP4P/2005)	308 ± 16	(6.56 ± 0.4) × 10^5^	15.5
*nanoMg*(TIP4P/2005)	1554 ± 90	(3.32 ± 0.2) × 10^6^	14.1
*microMg*(TIP4P-Ew)	660 ± 16	(1.41 ± 0.03) × 10^6^	15.0
*nanoMg*(TIP4P-Ew)	8618 ± 262	(1.84 ± 0.06) × 10^7^	12.6
*microMg*(TIP4P-D)	312 ± 8	(6.65 ± 0.2) × 10^5^	15.4
*nanoMg*(TIP4P-D)	1780 ± 20	(3.80 ± 0.05) × 10^6^	13.7
exp.	248,^74^ 314^73^	5.3 × 10^5^ from ref (73), 6.7 × 10^5^ from ref (74)	n.a.

a Number of transitions *N* in 1 μs for different Mg^2+^ parameters in 1 M MgCl_2_ solutions. The experimental value^73,74^ is obtained from eq S3. The rate constant *k* is calculated from the number of transitions *N* for *microMg* and *nanoMg* and eq S3. The errors for *N* and *k* are obtained from block averaging. ΔF* is the free energy difference between the top and the first minimum (Figure 4B). Values for parameters in TIP3P are taken from ref (26).

The improvement of the exchange rate compared to the standard sets from the literature is shown exemplary for SPC/E in Figure 4B (for the other waters, see Figure S5). The barrier height between the two stable states (i) and (ii), which corresponds to replacing one water molecule in the first hydration shell with a water molecule from the second shell (green and blue water molecules shown in Figure 4C), differs by more than 10 *k*_B_*T* for different force fields (Figure 4B). The different barrier heights provide a qualitative explanation of the different water exchange rates obtained with different force fields from the literature. Force fields with slow exchange kinetics (Åquist,^15^ Mamatkulov–Netz,^20^ and Li–Merz^21^) have high free energy barriers while force fields with a more attractive and long-ranged interaction potential (*microMg* and Li–Merz[12-6-4]^22^) have lower energetic barriers and yield exchange rates close to the experimental result. Finally, *nanoMg* has the lowest barrier, resulting in the fastest exchange kinetics that is particularly useful for enhanced sampling of Mg^2+^ binding, as will be discussed further below.

The microscopic mechanism of water exchange is, however, more complex than the one-dimensional free energy profiles suggest. The exchange involves the concerted motion of two water molecules^28,45^ and is captured more realistically by two-dimensional free energy profiles (Figures S6 and S7).

In addition, the self-diffusion coefficients *D*_0_ were calculated for each water model. Unlike the results in TIP3P water, where the diffusion coefficient matched the experimental value without optimization, *D*_0_ is underestimated for all water models investigated in the present study (Table 3). Likely, the better agreement in TIP3P is due to the higher self-diffusion constant of TIP3P.^36^

### 3.4 Optimization of the Activity Derivative

**Figure 5:**
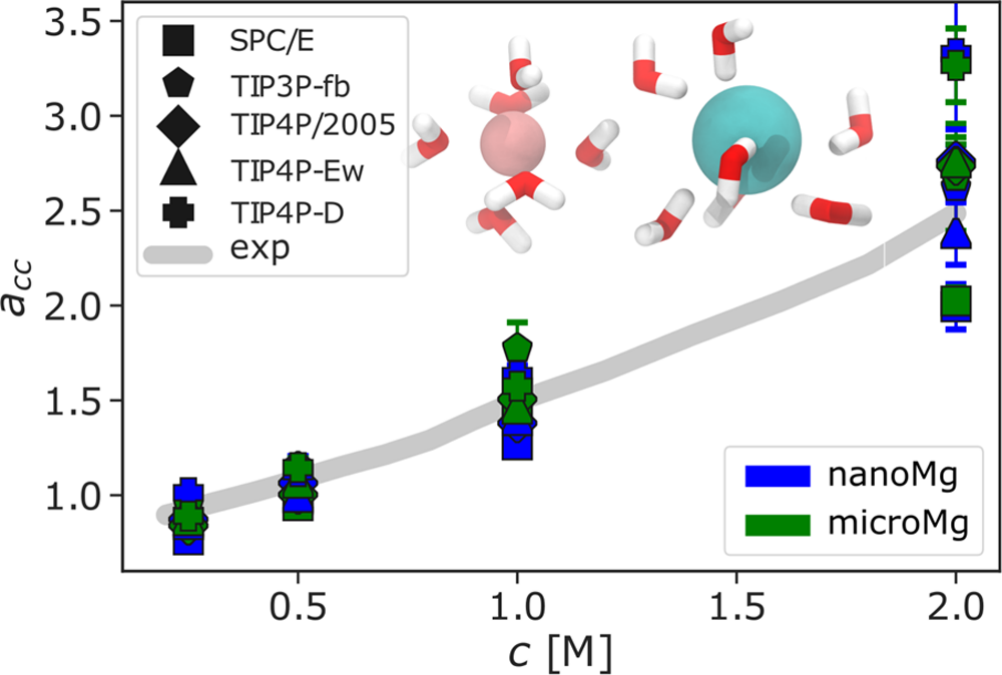
Optimization of ion–ion interactions. Activity derivative *a*_cc_ as a function of the MgCl_2_ salt concentration with the optimized scaling factors for *microMg* and *nanoMg* of the various water models (SPC/E,^46^ TIP3P-fb,^47^ TIP4P/2005,^48^ TIP4P-Ew,^49^ and TIP4P-D^50^) (Table 1) and from experiments.^88^ The inset shows a representative snapshot of Mg^2+^ in interaction with Cl^–^, including their first hydration shell. This snapshot was taken using *microMg*(SPC/E) and Cl^–^ parameters from Smith–Dang.^54^

Subsequently, we balance ion–water and ion–ion interactions by matching with experimental activity derivatives.^34,35,53,75,76^ Similarly to previous work,^20,23,25,26,35^ the standard combination rules (eq 2) (with λ_σ,ε_^Cl–^ = 1.0) overestimate the anion–cation interaction and consequently underestimate the activity derivative over the full parameter range. Since the standard combination rules are valid only in idealized cases, polarization and charge transfer can lead to deviations. Introducing scaling factors in the combination rule allows us to take these effects into account and to provide closer agreement with experimental results without changing Δ*G*_solv_, *R*_1_, *n*_1_, *D*_0_, or *k*. In all cases, increasing the effective size, σ_MgCl_, and decreasing the cation–anion interaction energy, ε_MgCl_, reproduces the experimental activity coefficient derivative of MgCl_2_ solutions over a broad concentration range (Figure 5). In particular, we found one set of universal scaling factors (λ_σ_^Cl^ = 1.59 and λ_ε_^Cl^ = 0.1) that leads to reasonable agreement with the experimental values for all five water models (Table 3, Figure 5). Note, however, that, at high concentrations, individually adjusted combination rules for water models such as TIP4P-D or SPC/E might lead to better agreement.

### 3.5 Optimization of the Binding Affinity to Nucleic Acids

So far, the optimization was based only on bulk ion properties. However, the transferability of such bulk-optimized ion parameters to describe the interactions of Mg^2+^ with biomolecules turned out to be limited.^25,26,30^ Therefore, in a last step, we optimized the interaction between Mg^2+^ and nucleic acids by matching with the experimental binding affinity and distance to one of the nonbridging phosphate oxygens.^77,78^ Similar to previous work,^19,26,30,57,58^ a dimethylphosphate (DMP) is used to mimic the phosphate backbone, the most important inner-sphere Mg^2+^ binding sites on larger RNA molecules.^5,77,79−81^ Here, the standard combination rules (eq 2 with λ_σ,ε_^RNA^ = 1.0) significantly overestimate the binding affinity toward the phosphate oxygen reflecting that the Mg^2+^–RNA interactions are too attractive (data not shown). As before, this problem can be solved by increasing the effective diameter via λ_σ_^RNA^ and simultaneously reducing the interaction energy via λ_ε_^RNA^ (Table 1). Note that, in this case, the scaling parameters for the different water models are similar but not identical since, unlike for chloride, the force field parameters of the RNA were not adjusted to the different water models.

**Figure 6:**
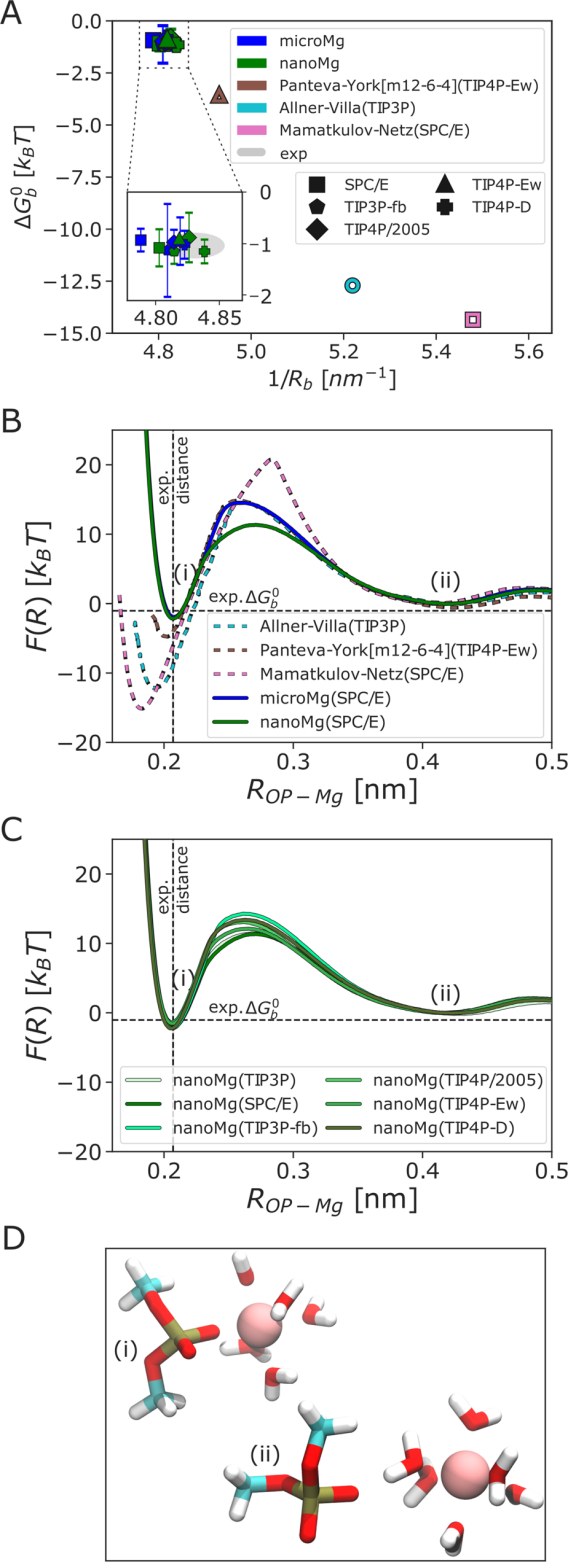
Optimization of ion–RNA interactions. (A) Binding affinity Δ*G*_b_^0^ in correlation with the inverse of the Mg^2+^–phosphate oxygen distance 1/*R*_b_ for *microMg* and *nanoMg* in the water models SPC/E,^46^ TIP3P-fb,^47^ TIP4P/2005,^48^ TIP4P-Ew,^49^ or TIP4P-D.^50^ The inset is a zoomed in view of the experimental area. The experimental values (gray) are taken from refs (77 and 78). (B, C) One dimensional free energy profiles along the distance between one of the nonbridging phosphate oxygens of the dimethylphosphate (DMP) and the Mg^2+^. The free energy profiles from Allnér–Villa and Panteva–York were obtained from refs (19 and 30) with permission. (D) Representative snapshot of the DMP in interaction with Mg^2+^, including the first hydration shell of Mg^2+^, in (i) inner-sphere and in (ii) outer-sphere contact. Snapshots were taken using *microMg*(SPC/E).

Figure 6 shows the binding affinity Δ*G*_b_^0^ and binding distance *R*_b_ for different water models and force field parameters from the literature.^19,20,22,30^ Clearly, the bulk-optimized parameters (Mamatkulov–Netz(SPC/E), Allnér–Villa(TIP3P)) significantly overestimate Δ*G*_b_^0^ and underestimate *R*_b_. The Panteva–York[m12-6-4] parameters^30^ provide improvement. However, in the optimization the 4-fold access of the phosphate oxygen binding site on the backbone compared to the nucleobase binding sites^77^ was taken into account, which is only applicable in the context of the modular model used in the experimental study. The affinity is thus overrated. Also, the binding distance *R*_b_ was not explicitly considered. With the optimized ion-RNA scaling factors for *microMg* and *nanoMg*, the results for Δ*G*_b_^0^ and *R*_b_ agree within errors with the experimental results for all 5 water models (Figure 6, Table 5, and Tables S9 and S10).

**Table 5:** Results for Ion–RNA Properties for the Optimized Parameters in Direct Comparison with Experimental Binding Affinity Towards the Phosphate Oxygen of DMP, Δ*G*_b_^0^, and Mg^2+^–Phosphate Oxygen Distance in Inner-Sphere Conformation, *R*_b_^a^

	Δ*G*_b_^0^ [*k*_B_*T*]	*R*_b_ [nm]	ΔF* [*k*_B_*T*]
*microMg*(TIP3P)^26^	–0.633 ± 0.6	0.207 ± 0.004	17.2
*nanoMg*(TIP3P)^26^	–0.375 ± 0.1	0.207 ± 0.004	13.6
*microMg*(SPC/E)	–1.26 ± 0.62	0.209 ± 0.004	16.5
*nanoMg*(SPC/E)	–1.19 ± 0.50	0.208 ± 0.004	13.5
*microMg*(TIP3P-fb)	–1.13 ± 0.90	0.208 ± 0.004	17.6
*nanoMg*(TIP3P-fb)	–1.15 ± 0.24	0.208 ± 0.004	16.5
*microMg*(TIP4P/2005)	–0.97 ± 0.24	0.208 ± 0.004	15.5
*nanoMg*(TIP4P/2005)	–0.88 ± 0.48	0.207 ± 0.004	14.5
*microMg*(TIP4P-Ew)	–0.95 ± 0.46	0.208 ± 0.004	15.3
*nanoMg*(TIP4P-Ew)	–0.91 ± 0.32	0.208 ± 0.004	13.7
*microMg*(TIP4P-D)	–1.02 ± 0.27	0.207 ± 0.004	16.4
*nanoMg*(TIP4P-D)	–1.15 ± 0.23	0.207 ± 0.004	15.6
exp.	–1.036^77^	0.206–0.208^78^	n.a.

a Δ*G*_b_^0^ is derived from the log stability constant of the DMP (log *K* = 0.45) given in ref (77). Barrier heights ΔF* of *microMg* and *nanoMg* separate the first and second minima of the free energy profile along the distance between one of the nonbridging phosphate oxygens of the DMP and Mg^2+^. ΔF* is the free energy difference between the top and the first minimum (Figure 6B,C). Values for usage in TIP3P are taken from ref (26).

The Mg^2+^ force field parameters and the water model have a significant influence on the ligand exchange kinetics.^45^ The effect is illustrated by the one-dimensional free-energy profiles as a function of the distance between Mg^2+^ and the phosphate oxygen for different force fields and water models (Figure 6B,C). The free energy profiles show two stable states (i) and (ii), corresponding to the inner-sphere conformation (direct contact between Mg^2+^ and phosphate oxygen) and the water-mediated outer-sphere conformation (Figure 6D). The bulk-optimized parameters (Mamatkulov–Netz(SPC/E), Allnér–Villa(TIP3P)) significantly overestimate Δ*G*_b_^0^ and underestimate *R*_b_ as reflected by the shift and depth of the first minimum (Figure 6B). For *microMg* and *nanoMg*, the inner-sphere and outer-sphere minima are identical, reflecting that the parameters have identical thermodynamic and structural properties. In addition, clear differences are observed for the barrier height, which corresponds in large part to the free energy cost of exchanging one water molecule from the first hydration shell with a water from the second shell. Force fields that underestimate the rate of water exchange (Mamatkulov–Netz(SPC/E), Allnér–Villa(TIP3P)) have high energetic barriers, Mg^2+^ association/dissociation too slow, binding affinity too high, and binding distance too small (Figure 6B). *MicroMg* and *nanoMg* provide significant improvement (Table 5). At the same time, the free energy barrier for *nanoMg* is up to 4 *k*_B_*T* lower compared to *microMg* depending on the water models used (Figure 6B, Table 5).

Reproducing Δ*G*_b_^0^ and *R*_b_ is crucial to correctly describe the structure and thermodynamics of specifically bound Mg^2+^ ions. Since *microMg* and *nanoMg* reproduce the properties of specifically bound ion as well as important bulk properties, both parameter sets are equally suited to calculate the distribution of Mg^2+^ around biomolecules. In simulations that target the binding kinetics of the metal cations, the *microMg* parameter set should be used. To enhance the sampling of binding events, the *nanoMg* set can be used since the exchange kinetics is accelerated, thereby improving the sampling. However, the acceleration of the binding kinetics strongly depends on the water model used. For the largest speed-up, we recommend using *nanoMg* in combination with the TIP3P or SPC/E water model (Figure 4A).

## 4 Conclusions

The distinct role of magnesium in biological systems has driven the development of force fields for molecular dynamics simulations. Despite considerable efforts, Mg^2+^ force fields based on the 12–6 Lennard-Jones (LJ) potentials showed significant shortcomings in reproducing a broad range of structural, thermodynamic, and kinetic properties. Since Mg^2+^ strongly polarizes its environment, the lack of polarizability and charge transfer effects in classical simulations likely causes such deviations. Recently, progress was made by an optimization procedure that implicitly accounts for polarizability. Considering an enlarged range of possible LJ parameters for Mg^2+^–water interactions and optimizing the Lorentz–Berthelot combination rule for Mg^2+^–Cl^–^ and Mg^2+^–RNA interactions yielded simple and accurate force fields in TIP3P water.^26^

However, a large variety of water models exist, and models with improved properties are increasingly used in biomolecular simulations. Since simulations rely on available water models and ion force fields that provide physically meaningful results when combined,^82^ the transferability of the Mg^2+^ parameters to other water models needs to be assessed and the parameters need to be optimized if necessary. Our results reveal that the Mg^2+^ parameters developed in combination with TIP3P show limited transferability to SPC/E, TIP3P-fb, TIP4P/2005, TIP4P-Ew, and TIP4P-D. While for the 3-site water models (TIP3P-fb and SPC/E) the deviations from the experimental solvation free energy are small, significant deviations are observed for the 4-site models (TIP4P/2005, TIP4P-Ew, and TIP4P-D).

To provide improvement, we have systematically developed improved Mg^2+^ parameters. The optimized parameters (Table 1) reproduce the solvation free energy, the distance to oxygens in the first hydration shell, the hydration number, the activity coefficient derivative in MgCl_2_ solutions, and the binding affinity and distance to the phosphate oxygens on nucleic acids (Tables 3, 5). In order to provide consistent and robust results for the solvation free energy and the activity derivative, Cl^–^ was chosen as a reference ion and its parameters were optimized in a preceding step for each water model (Table 2). Here, an alternative and promising approach for future work is the simultaneous optimization of anion and cations parameters, which eliminates the necessity to select a reference ion.^83^ In addition, including experimental binding affinities toward specific ion binding sites on biomolecules has turned out to be essential to capture the structure of specifically bound ions^26^ as well as to reproduce the structural properties of large nucleic acids.^42^

Two parameter sets are presented for each water model: The first sets, *microMg*, yield water exchange on the microsecond time scale in agreement with experimental results.^73,74^ For the second sets, *nanoMg*, the parameters were chosen to yield the highest exchange rate possible while still reproducing all other thermodynamic and structural properties. Since the water models have different properties,^36^ including different dielectric constants and diffusion coefficients, the maximum achievable exchange rate differs and ranges from 10^6^ to 10^8^ exchanges per second (Table 4).

In summary, the Mg^2+^ parameters presented here provide simple, computationally efficient, and highly accurate models for the simulation of Mg^2+^ ions in aqueous solutions of SPC/E, TIP3P-fb, TIP4P/2005, TIP4P-Ew, and TIP4P-D water. For simulations targeting the kinetics of ion pairing or ion binding, the *microMg* is recommended. For simulations targeting the distribution of Mg^2+^ around nucleic acids, proteins, or lipids the *nanoMg* is recommended to enhance the sampling of binding events. Here, the parameters for TIP3P^26^ or SPC/E yield the largest enhancement.

## Supporting Information Available

Additional methods, including detailed lists on the different setups used in this study, and additional results: single-ion properties and their isosurfaces calculated to study the transferability of the original parameters *microMg*(TIP3P) and *nanoMg*(TIP3P) to other water models; additional Lennard-Jones interaction potentials and one-dimensional free energy profiles of the optimized parameters for TIP3P-fb, TIP4P/2005, TIP4P-Ew, and TIP4P-D that are not shown in the main text; two-dimensional free energy profiles of all 10 optimized Mg^2+^ parameter sets; and binding affinity values obtained from alchemical transformation for all 10 optimized parameter sets (PDF)
