# Supplementary material for: Optimized Magnesium Force Field Parameters for Biomolecular Simulations with Accurate Solvation, Ion-Binding, and Water-Exchange Properties in SPC/E, TIP3P-fb, TIP4P/2005, TIP4P-Ew, and TIP4P-D
Source: J Chem Theory Comput. 2021 Dec 9;18(1):526–37. doi: 10.1021/acs.jctc.1c00791 (PMC8757469; doi:10.1021/acs.jctc.1c00791)
Supplement: Supplementary file 1 — ct1c00791_si_001.pdf [file ct1c00791_si_001.pdf]

Supplementary Information: Systematic Optimization of Magnesium Force Field Parameters for Biomolecular Simulations with Accurate Solvation, Ion-pairing, and Water Exchange Properties in SPC/E, TIP3P-fb, TIP4P/2005, TIP4P-Ew, and TIP4P-D.

Kara K. Grotz and Nadine Schwierz*

Department of Theoretical Biophysics, Max-Planck-Institute of Biophysics, Frankfurt am Main, Germany.

E-mail: nadine.schwierz@biophys.mpg.de

Contents

S1 Supplementary methods **S3**

S1.1 Literature values for central Mg^{2+} properties . . . S3

S1.2 Water models . . . S3

S1.3 Simulation setups . . . S4

S1.4 Diffusion coefficient . . . S5

S1.5 Rate constant of water exchange . . . S5

S1.6 Calculation of activity derivatives . . . S6

S1.7 Calculation of free energy profiles . . . S7

S1.8 Binding affinity towards DMP . . . S9

S1.8.1 General strategies for computing binding affinities . . . S10

S1.8.2 Methods in this work for computing binding affinities . . . S11

S2 Supplementary results **S12**

S2.1 Transferability . . . S12

S2.2 Isosurfaces S13

S2.3 Lennard-Jones interaction potentials S14

S2.4 One-dimensional free energy profiles for Mg^{2+}-water interactions S15

S2.5 Two-dimensional free energy profiles for Mg^{2+}-water interactions S16

S2.6 Binding affinities S18

S3 Bibliography **S20**

S1 Supplementary methods

S1.1 Literature values for central Mg^{2+} properties

Previous force fields for Mg^{2+} failed to provide a quantitative description of central properties of the ion. In Table S1 the central properties are listed for Mg^{2+} as obtained from the literature and compared to experiments.

Table S1: Values for Mg^{2+} models from the literature for central properties of the ion in comparison to experiments and one model of the current work.

	R_1 [nm]	ΔG_{solv} [kJ/mol]	k [s^{-1}]	$\Delta G_{\mathrm{b}}^{0}$ [$\mathrm{k_B T}$]
Mamatkulov-Schwierz (TIP3P) [1]	0.196 [1]	-2531.1 [1]	24 ± 9 [2]	-14.53 ± 0.95 [3]
Allner-Villa (TIP3P) [4]	0.204 [4]	-2397.3 [5]	$\ll 2.39 \times 10^5$ [6]	-12.17 ± 0.14 [6]
Li-Merz (TIP3P) [7]	0.194 [7]	-2527.0 [7]	$\ll 3.52 \times 10^4$ [6]	n.a.
Li-Merz (TIP3P) [12-6-4] [8]	0.208 [8]	-2519.1 [8]	$(1.44 \pm 0.03) \times 10^7$ [6]	n.a.
Mamatkulov-Netz (SPC/E) [9]	0.196 [9]	-2532.0 [9]	n.a.	-14.35 ± 0.48
Aquist (SPC/E) [10]	0.199 [5]	-2505.3 [5]	n.a.	n.a.
Li-Merz (SPC/E) [7]	0.195 [7]	-2521.2 [7]	n.a.	n.a.
Li-Merz (SPC/E) [12-6-4] [8]	0.208 [8]	-2524.9 [8]	n.a.	n.a.
Li-Merz (TIP4P-Ew) [7]	0.199 [7]	-2520.3 [7]	n.a.	n.a.
Li-Merz (TIP4P-Ew) [12-6-4] [8]	0.208 [8]	-2526.2 [8]	n.a.	-7.65 ± 0.35 [6]
microMg (SPC/E) [current work]	0.209	-2530.5	$(9.62 \pm 1) \times 10^5$	-1.266 ± 0.62
Exp.	0.209 ± 0.004 [11]	-2532 [12]	$5.3 - 6.7 \times 10^5$ [13,14]	-1.036 [15]

S1.2 Water models

We used five different rigid water models and listed their partial charges and Lennard-Jones parameters in Table S2. For SPC/E, the bond length is fixed at 0.1 nm and the bond angle at 109.47°. For TIP3P-fb, the bond length is fixed at about 0.10118 nm and the bond angle at about 108.15°. Otherwise, the bond length is 0.09572 nm and the bond angle is 104.52°.

Table S2: Parameters of different water models. The 3-site waters (SPC/E, TIP3P-fb) carry their negative partial charge ($q_{\mathrm{Ow/Mw}}$) on the oxygen Ow, whereas for the 4-site waters (TIP4P/2005, TIP4P-Ew, and TIP4P-D), it is located on the slightly shifted additional site Mw. q_{H} is the partial charge of the hydrogen atoms and σ_{Ow} and $\varepsilon_{\mathrm{Ow}}$ the Lennard-Jones parameters of the water oxygen.

	$q_{\mathrm{Ow/Mw}}$ [e]	q_{H} [e]	σ_{Ow} [nm]	$\varepsilon_{\mathrm{Ow}}$ [kJ/mol]
SPC/E	-0.8476	0.4238	0.3166	0.650
TIP3P-fb	-0.848448	0.424224	0.317796456355	0.652143528104
TIP4P/2005	-1.1128	0.5564	0.31589	0.774898
TIP4P-Ew	-1.04844	0.52422	0.316435	0.680946
TIP4P-D	-1.16	0.58	0.316508	0.936256

S1.3 Simulation setups

In all simulations long-range electrostatic forces were handled applying the particle-mesh Ewald method [16] with periodic boundary conditions, a Fourier spacing of 0.12 nm and a grid interpolation up to order 4. Short-range Coulomb and Lennard-Jones interactions were cut off at 1.2 nm. In all simulations, a 2 fs timestep was used. In all simulations, ions and rigid water models are used, in those with AMBER we employed SETTLE, [17] and in those with GROMACS we employed LINCS. [18] Hydrogen bonds are constrained in all simulations containing DMP using LINCS. All the simulations started with an energy minimization by steepest decent, which was followed by two equilibration steps of 0.5 ns in NVT and 1 ns in NPT. The temperature was kept at 300 K and the pressure at 1 atm using thermostat and barostat of Berendsen. [19] Position restraints were applied to all ions to ensure that no ions are pairing before hydration shells can properly form. The restraints were released in the production run. Lorentz-Berthelot combination rules were chosen for all simulations. The simulations to compute solvation free energy ΔG_{solv}, radius of the first hydration shell R_1, and coordination number n_1, were performed employing the thermostat and barostat of Berendsen [19] with $\tau = 0.1$ and $\tau_{\mathrm{p}} = 1.0$ to ensure a temperature of 300 K and roughly an atmospheric pressure of 1 bar. All other simulations were performed with the velocity rescaling thermostat by Bussi et al. [20] with $\tau = 0.1$. In case of the simulations in isothermal-isobaric ensemble (NPT in Table S3 and S4) the Parrinello-Rahman barostat [21] with $\tau_{\mathrm{p}} = 5.0$ was applied. Frames were written out every 0.2 ps if not stated otherwise. For both engines GROMACS and AMBER, the same setups were employed.

Table S3: This table shows all simulation setups of the 3-site water models SPC/E and TIP3P-fb. In 'Phys. property', the physical properties are listed that we obtained from the respective simulation using the respective 'Method' (FEP: free energy perturbation, unbiased: straight forward simulations without additional biases, US: umbrella sampling). 'System' lists all particles of the respective simulation and 'Duration' their duration (products show for FEP and US simulations the individual simulation windows). 'L' indicates the simulation box size (edge length) for (cu) cubic and (do) rhombic dodecahedron box shapes. 'Ensemble' expresses if an canonical ensemble (with constant number of particles N, constant volume V, and constant temperature T), or an isobaric-isothermal ensemble (with constant pressure P and flexible simulation box size) was used. [GMX and AMBER specify the setup used for the 12-6 based (GMX) or the 12-6-4 based parameters (AMBER)].

Phys. property	Method	System	Duration	L	Ensemble
ΔG_{solv}, R_1, n_1	FEP	1 Mg^{2+}, 506 water	40 × 1 ns	2.5 nm (cu)	NPT
D_0	unbiased	1 Mg^{2+}, 506 water	100 ns	2.5 nm (cu)	NVT
a_{cc}, k	unbiased	39 Mg^{2+}, 78 Cl^-, 2048 water (1 M)	1 μs	4 nm (cu)	NPT
a_{cc}	unbiased	73 Mg^{2+}, 146 Cl^-, 1961 water (2 M)	150 ns	4 nm (cu)	NPT
a_{cc}	unbiased	40 Mg^{2+}, 20 Cl^-, 2105 water (0.5 M)	150 ns	4 nm (cu)	NPT
a_{cc}	unbiased	20 Mg^{2+}, 10 Cl^-, 2135 water (0.25 M)	150 ns	4 nm (cu)	NPT
$F(r_{\mathrm{MgOw}})$	US (GMX)	1 Mg^{2+}, 505 water	68 × 3 ns	2.5 nm (cu)	NPT
$F(r_{\mathrm{MgOw}})$	US (AMBER)	1 Mg^{2+}, 500 water	68 × 3 ns	2.5 nm (cu)	NPT
$F(r_{\mathrm{MgOw_1}}, r_{\mathrm{MgOw_2}})$	US	1 Mg^{2+}, 506 water	946 × 5 ns	2.5 nm (cu)	NVT
ΔG^0_{b}	FEP	1 DMP, 1 Mg^{2+}, 1 Cl^-, 1492 water	20 × 15 ns	4 nm (do)	NPT
ΔG^0_{b}, $F(r_{\mathrm{MgOP}})$	US	1 DMP, 1 Mg^{2+}, 1 Cl^-, 1492 water,	67 × 20 ns	4 nm (do)	NVT
$\Delta G^0_{\mathrm{ref}}$, $F(r_{\mathrm{ref-OP}})$	US	1 DMP, 1 ref atom, 1 Cl^-, 1492 water	67 × 20 ns	4 nm (do)	NVT

Table S4: This table lists all simulation setups using the 4-site water models TIP4P/2005, TIP4P-Ew, and TIP4P-D that differ from Table S4. If the required 'Phys. property' is not listed here, the same setup is applied as in Table S3.

Phys. property	Method	System	Duration	L	Ensemble
a_{cc}, k	unbiased	39 Mg^{2+}, 78 Cl^-, 2055 water (1 M)	150 ns	4 nm (cu)	NPT
a_{cc}	unbiased	73 Mg^{2+}, 146 Cl^-, 1909 water (2 M)	150 ns	4 nm (cu)	NPT
a_{cc}	unbiased	20 Mg^{2+}, 40 Cl^-, 2135 water (0.5 M)	150 ns	4 nm (cu)	NPT
a_{cc}	unbiased	10 Mg^{2+}, 20 Cl^-, 2171 water (0.25 M)	150 ns	4 nm (cu)	NPT
ΔG_b^0	FEP	1 DMP, 1 Mg^{2+}, 1 Cl^-, 1536 water	20 × 15 ns	4 nm (do)	NPT
ΔG_b^0, $F(r_{\mathrm{MgOP}})$	US	1 DMP, 1 Mg^{2+}, 1 Cl^-, 1536 water	67 × 20 ns	4 nm (do)	NVT
$\Delta G_{\mathrm{ref}}^0$, $F(r_{\mathrm{ref-OP}})$	US	1 DMP, 1 ref atom, 1 Cl^-, 1536 water	67 × 20 ns	4 nm (do)	NVT

S1.4 Diffusion coefficient

Diffusion coefficients D_0 were calculated from 10 ns long NVT simulations. The first 1 ns was excluded in each trajectory from the analysis. We calculated the coefficients $D_{\mathrm{pbc}}(L)$ from a straight line fit of the slope of the mean-squared displacement of the single ion and took only the linear part into account. The obtained diffusion coefficient was size-corrected[22] by

$$D_0 = \frac{\eta_{\mathrm{W}}}{\eta_{\mathrm{water}}} \left[D_{\mathrm{pbc}}(L) + \frac{k_B T \zeta_{\mathrm{ew}} \alpha}{6\pi\eta L} \right]. \tag{S1}$$

We explicitly took the different viscosities η_{W} of the different water models W into account (Table S5).

Table S5: Viscosities η_{W} for each water model W. Values for SPC/E and TIP4P/2005 are taken from ref.,[23] the value for TIP4P-D is taken from ref.,[24] the other values are from their original publications (TIP3P-fb,[25] TIP4P-Ew[26]).

	[kg m^{-1} s^{-1}]
$\eta_{\mathrm{SPC/E}}$	$7.29 \cdot 10^{-4}$
$\eta_{\mathrm{TIP3P\text{-}fb}}$	$3.13 \cdot 10^{-4}$
$\eta_{\mathrm{TIP4P/2005}}$	$8.55 \cdot 10^{-4}$
$\eta_{\mathrm{TIP4P\text{-}Ew}}$	$7.2 \cdot 10^{-4}$
$\eta_{\mathrm{TIP4P\text{-}D}}$	$9.37 \cdot 10^{-4}$

S1.5 Rate constant of water exchange

The most popular theory to calculate reaction rates is transition state theory (TST).[27,28] In simple systems for which the reaction coordinate is exactly known, TST gives an accurate estimate of the rate. However, in complex many body systems as the ones presented here, TST can fail due to the violation of the non-recrossing hypothesis, one of the fundamentals of the theory. Therefore, in the following an alternative approach is presented (for more details see ref.[6]).

For the exchange of water from the first hydration shell of Mg^{2+}, the rate constant k is defined by[13]

$$\text{rate} = 6 \cdot k \cdot [\text{Mg}(\text{H}_2\text{O})_6^{2+}] \ , \tag{S2}$$

where 6 is the coordination number of the first hydration shell and $[\text{Mg}(\text{H}_2\text{O})_6^{2+}]$ is the concentration of hexa-coordinated Mg^{2+} ions.

In this work, the water exchange rate constant k is calculated by counting the total number of transitions that are observed within a 1 μs long trajectories of a 1 M $MgCl_2$ solution. As a transition N, we regard the exchange of waters between the first and second hydration shell of Magnesium (the exchange from first to second hydration shell and the reverse transition are counted as individual events). The water exchange rate constant k is hence given by

$$k = \frac{1}{N_{\text{H}_2\text{O}}} \cdot \frac{N}{2 \cdot t_{\text{B}}} \ , \tag{S3}$$

where $N_{\text{H}_2\text{O}}$ is the number of water molecules in the simulation box. $t_B = N_{\text{Mg}} \cdot p_{\text{B}} \cdot t_{\text{sim}}$ is the cumulative time the water molecule spends in the first hydration shell of any Mg^{2+} ion. N_{Mg} is the number of Mg^{2+} ions in the simulation box, $p_{\text{B}} = 6/(N_{\text{H}_2\text{O}} - 6)$ is the probability of water to be in the first hydration shell and t_{sim} is the total simulation time. Errors are calculated from block averaging[29] by dividing the trajectory into 2 blocks.

S1.6 Calculation of activity derivatives

The derivative a_{cc} of the activity $a_{\text{c}} = \rho_{\text{c}} y_{\text{c}}$ with activity coefficient y_{c} is defined via

$$a_{\text{cc}} = \left(\frac{\partial \ln a_{\text{c}}}{\partial \ln \rho_{\text{c}}}\right)_{\text{P,T}} = 1 - \left(\frac{\partial \ln y_{\text{c}}}{\partial \ln \rho_{\text{c}}}\right)_{\text{P,T}} = \frac{1}{1 + \rho_{\text{c}}(G_{\text{cc}} - G_{\text{co}})} \ , \tag{S4}$$

with respect to the natural logarithm of the number density ρ_{c}. The expressions G_{cc} and G_{co} are for divalent cations obtained[30,31] from

$$G_{\text{cc}} = \frac{1}{9}\Big[G_{++} + 4(G_{--} + G_{+-})\Big] \tag{S5}$$

and

$$G_{\text{co}} = G_{\text{oc}} = \frac{1}{3}G_{+\text{o}} + \frac{2}{3}G_{-\text{o}} \ , \tag{S6}$$

where G_{ij} are the Kirkwood-Buff (KB) integrals[31–33] (eq S7) with +, −, and o denoting the cation, anion, and water oxygen, respectively. To obtain these KB integrals we followed that same strategy as in ref.,[6]

$$G_{ij} = 4\pi \int_0^\infty \left[g_{ij}^{\mu\mathrm{VT}}(r_{ij}) - 1\right] r_{ij}^2 dr_{ij} \ , \tag{S7}$$

where $g_{ij}^{\mu\mathrm{VT}}(r_{ij})$ is the radial distribution function of the two species in the grand canonical ensemble, with r_{ij} being the center of mass distance between the two. Note that the simulation were done in the NPT ensemble. Therefore, we introduce a correction factor such that the radial distribution function used in the calculations of the KB integrals shows the correct asymptotic behavior at large distances (for more details see refs.[1,6,31,33]).

S1.7 Calculation of free energy profiles

We computed all free energy profiles $F(R)$ with umbrella sampling[34,35] and the weighted histogram analysis method (WHAM)[36] to combine the individual umbrella windows.

1D $\mathbf{Mg^{2+}}$ - water: One dimensional free energy profiles as a function of the distance between Mg^{2+} and the oxygen atom of the leaving water molecule for the 12-6 type force fields were computed with GROMACS[37] (version 2020). The profile of the 12-6-4 type force field in SPC/E water was computed using AMBER[38] (version 2018) and PLUMED[39] (version 2.5). Force constants and window spacing were in both setups $k = 400,000$ kJ/(mol nm^2) and 0.005 nm [$0.17 \leq R_{\mathrm{MgOx}} < 0.4$ nm] and $k = 100,000$ kJ/(mol nm^2) and 0.01 nm [$0.4 \leq R_{\mathrm{MgOx}} < 0.6$ nm]. Frames for the analysis were considered every 0.5 ps. We took a bin width of 5.4 x 10^{-4} nm into account for WHAM.

To ensure convergence, windows of size 50 ns were obtained and subdivided into blocks of (A) 10 x 5 ns, (B) 5 x 10 ns, and (C) 2 x 25 ns for the Mamatkulov-Netz parameters in SPC/E water (Figure S1). Of each individual block a free energy profile is calculated. Afterwards, we transformed the free energy profiles into potentials of mean force $V^{\mathrm{PMF}}(R)$ by applying a Jacobian correction,

$$V^{\mathrm{PMF}}(R) = F(R) + 2k_{\mathrm{B}}T \ln R, \tag{S8}$$

to take radial distances rather the Cartesian coordinates into account. We find that with windows of duration (A) 5 ns and (B) 10 ns the individual profiles show deviations whereas with a duration of (C) 25 ns the results are converged.

All other water exchange free energy profiles were thus simulated for 25 ns (see Table S3 and S4 for more

details on the simulation setups).

As an additional validation step, we obtained the radial distribution function $g(R)$ from the 1 μs 1 M $MgCl_2$ trajectory and the free energy profile via Boltzmann inversion. Note that the free energy profile from the radial distribution function is used only to validate the depth of the two minima. The barrier height cannot be derived from this method as the transitions of water exchange are too rare. Further insight into the quality of the umbrella sampling method is provided by the evenly spaced and overlapping histograms (Figure S1D).

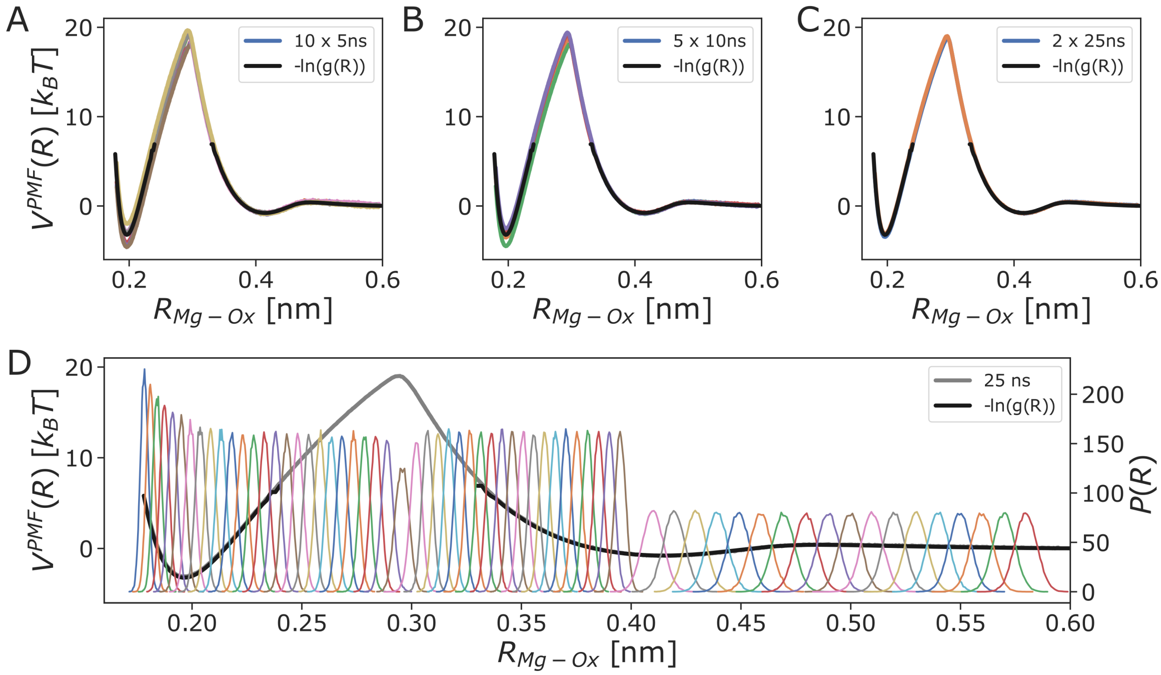

Figure S1: Convergence check. Potential of mean force $V^{\mathrm{PMF}}(R)$ along the distance between a Mg^{2+} ion and the oxygen atom of the leaving water molecule, obtained from windows of 50 ns that are subdivided into blocks of (A) 10 x 5 ns, (B) 5 x 10 ns, and (C) 2 x 25 ns and $V^{\mathrm{PMF}}(R)$ obtained from $-\ln g(R)$. In (D) additional to $V^{\mathrm{PMF}}(R)$ obtained from 25 ns long windows, the histograms are shown. Parameters used are Mamatkulov-Netz and SPC/E water.

1D Mg^{2+} - phosphate oxygen: The free energy profile along the distance between Mg^{2+} and one of the two non-bridging phosphate oxygens of the dimethylphosphate (DMP) was obtained with force constants and window spacing of $k = 300,000$ kJ/(mol nm^2) and 0.0075 nm [$0.15 \leq R_{\mathrm{MgOP}} < 0.525$ nm] and $k = 5,000$ kJ/(mol nm^2) and 0.02 nm [$0.525 \leq R_{\mathrm{MgOP}} < 0.885$ nm], respectively, using GROMACS[40] (version 2020). PLUMED[39] was employed to include an additional bias avoiding any direct interaction with any of the DMP atoms but the selected phosphate oxygen (see ref.[6] for more details). Frames for the analysis were considered every 0.5 ps. For WHAM a bin width of 9.2 x 10^{-4} nm was considered.

To ensure convergence, windows of size 100 ns were obtained and subdivided into blocks of (A) 10 x 10

ns, (B) 5 x 20 ns, and (C) 2 x 50 ns for the Mamatkulov-Netz SPC/E parameters (Figure S2). The results show that 20 ns per window are sufficient to provide converged results. For all other profiles for DMP and Mg^{2+}, windows were simulated for 20 ns (see Table S3 and S4 for more details on the simulation setups). Additionally, Figure S2D shows the overlapping histograms of neighboring simulation windows.

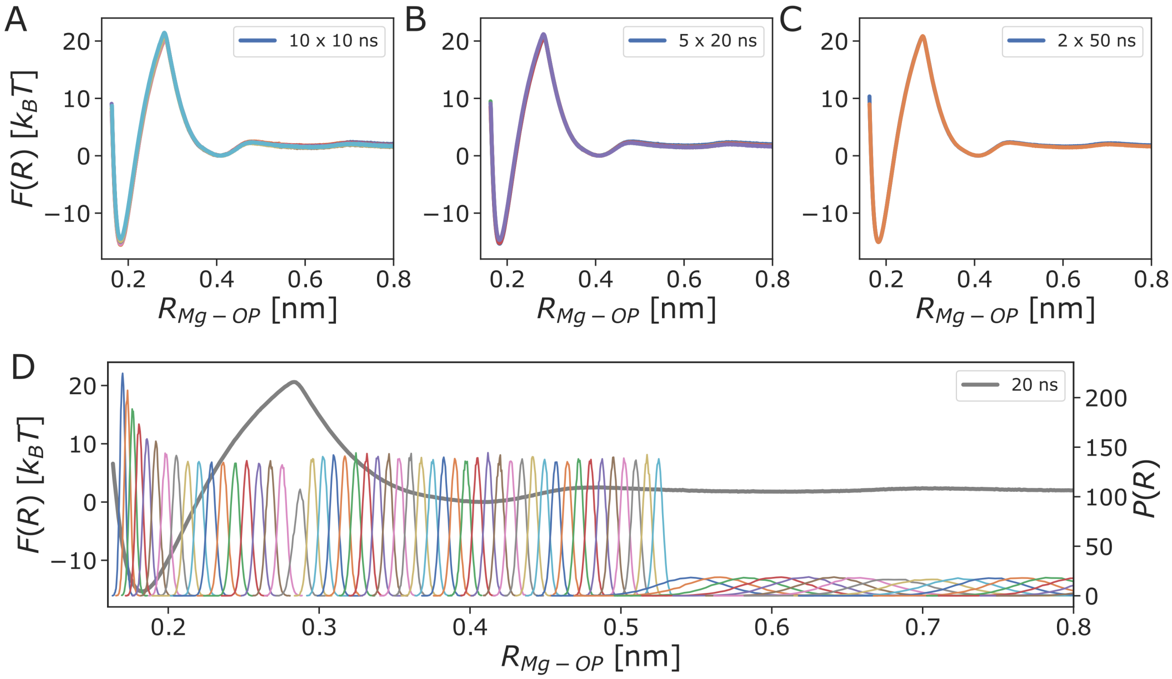

Figure S2: Convergence check. Free energy profile $F(R)$ along the distance between one of the non-bridging oxygens of the DMP and a Mg^{2+} ion, obtained from windows of 100 ns that are subdivided into blocks of (A) 10 x 10 ns, (B) 5 x 20 ns, and (C) 2 x 50 ns. In (D) additional to $F(R)$ obtained from 20 ns long windows, overlapping distance histograms are shown. Parameters used are Mamatkulov-Netz and SPC/E water.

S1.8 Binding affinity towards DMP

Calculating binding affinities from molecular dynamics simulations can be challenging since the results depend sensitively on accurate sampling. In principle, one can obtain the binding affinity of a metal cation toward the phosphate oxygen in three different ways: (i) Via association/dissociation rates, (ii) via free energy perturbation calculations, or (iii) via integrating potentials of mean force (PMFs). All three methods yield consistent results if accurately sampled (see Table S6 for results for Ca^{2+} from previous works). To avoid possible pitfalls, we strongly recommend to use different methods to verify binding affinities obtained by computer simulations.

S1.8.1 General strategies for computing binding affinities

Association/dissociation rates If association k_{on} and dissociation k_{off} rates are available, the free energy of binding can be calculated from

$$\Delta G_{\mathrm{b}}^{0} = -k_{\mathrm{B}}T \cdot \ln\left(\frac{c^{0}}{[M]} \cdot \frac{k_{\mathrm{on}}}{k_{\mathrm{off}}}\right), \tag{S9}$$

with respect to the standard concentration $c^0 = 1$ M. For Ca^{2+}, the rates were calculated in our previous work[41] and the results for the resulting free energy are shown in Table S6.

Free energy perturbation The binding free energy can be obtained from free energy perturbation using the double decoupling method (DDM)[42] or alchemical transformation calculations.[43,44] In the former (eq S10), both van der Waals as well as electrostatic interactions are decoupled for the ion in solution ($\Delta G^{\mathrm{solv}}_{\mathrm{elec+vdW}}$) and turned back on for an ion at the binding site ($\Delta G^{\mathrm{BS}}_{\mathrm{elec+vdW}}$). The effect of the restraints to keep the ion in the binding site during the decoupling in bulk ($\Delta G^{\mathrm{solv}}_{\mathrm{rest}}$) and in the binding site ($\Delta G^{\mathrm{BS}}_{\mathrm{rest}}$) has to be included[42]

$$\Delta G_{\mathrm{b}}^{0} = \Delta G^{\mathrm{solv}}_{\mathrm{elec+vdW}} + \Delta G^{\mathrm{BS}}_{\mathrm{elec+vdW}} + \Delta G^{\mathrm{solv}}_{\mathrm{rest}} + \Delta G^{\mathrm{BS}}_{\mathrm{rest}} \tag{S10}$$

In the alchemical transformation (eq S11), a reference ion of the same valency is used. The ion of interest is alchemically transformed into the reference ion, both in bulk ($\Delta G^{\mathrm{solv}}_{\mathrm{ion}\to\mathrm{ref}}$) as well as at the binding site ($\Delta G^{\mathrm{B}}_{\mathrm{ref}\to\mathrm{ion}}$)

$$\Delta G_{\mathrm{b}}^{0} = \Delta G^{\mathrm{solv}}_{\mathrm{ion}\to\mathrm{ref}} + \Delta G^{0}_{\mathrm{ref}} + \Delta G^{\mathrm{BS}}_{\mathrm{ref}\to\mathrm{ion}} \tag{S11}$$

where $\Delta G^{0}_{\mathrm{ref}}$ is the binding affinity of the reference ion. To check the convergence, the transformation is done in opposite directions (i.e. forward and backward). For Ca^{2+}, the values for both methods were obtained from our previous works[3,41] and are listed in Table S6.

Integration of a potential of mean force Here, the binding affinity is obtained by integrating the potential of mean force $V^{\mathrm{PMF}}(r)$ (eq S8) along the distance r between the binding site and the ion of interest,

$$\Delta G_{\mathrm{b}}^{0} = -k_{\mathrm{B}}T \cdot \ln\left(\frac{c^{0}}{[M]} \cdot \frac{\int_{0}^{\mathrm{r}^{\dagger}} r^{2} e^{-\mathrm{V}^{\mathrm{PMF}}(\mathrm{r})/\mathrm{k_B T}} dr}{\int_{\mathrm{r}^{\dagger}}^{\mathrm{r_L}} r^{2} e^{-\mathrm{V}^{\mathrm{PMF}}(\mathrm{r})/\mathrm{k_B T}} dr}\right), \tag{S12}$$

where $[M]$ is the ion concentration of the simulation box, r_{L} is the radius of a sphere that contains the same number of water molecules as the simulation box and $r^{\dagger}$ is the position of the maximum of the PMF. Note that both $[M]$ and r_{L} are dependent on the number of waters in the simulation such that eq S12 becomes

independent of the box size used. The value for Ca^{2+} listed in Table S6 is taken from our previous work.[6]

As shown in Table S6 the results for Ca^{2+} for all four methods match within error. This demonstrates that the methods are converged and that all of these setups to obtain the binding free energy for Ca^{2+} (which is used as reference ion in this current work) are sufficiently accurate.

Table S6: Comparison of different methods to compute the free binding energy of Ca^{2+} to a non-bridging phosphate oxygen using the parameters by Mamatkulov-Schwierz in TIP3P.[1]

Method	Formula	$\Delta G^0_{\mathrm{Ca}^{2+}}$ [k_BT]
Rates (TREMD)	(S9)	-4.51 ± 1.27 [41]
FEP DDM	(S10)	-4.57 ± 0.90 [41]
FEP alchem. trafo.	(S11)	-4.86 ± 0.85 [3]
PMF	(S12)	-4.84 ± 0.18 [6]

S1.8.2 Methods in this work for computing binding affinities

In this present work, to fine-tune the interaction and find scaling factors between the different Mg^{2+} models and the phosphate oxygen, we primarily computed the binding affinities via integration of PMFs (eq S12), as this strategy also includes a more mechanistic picture due to the obtained energetic profiles. The free energy profiles (most of which are shown in Figure 6B,C in the main text) were obtained using umbrella sampling. Details on all the simulation parameters are given in Section S1.2 and all parameters for the umbrella sampling in Section S1.6. (All simulation parameters are exactly the same as in our previous work before.[6])

The concentrations of Mg^{2+} in the simulation boxes used in eq S12 are $[M] = 0.037$ M and 0.036 M for the 3-site and 4-site waters, respectively. Errors are calculated by dividing the 20 ns long windows into 4 blocks, and subsequent calculation of PMFs and thus ΔG^0_b from each block and block averaging.

As an independent validation step, we selected a second method to obtain the binding affinities. Association/dissociation rates are very difficult to obtain for Mg^{2+} due to the long timescales involved. Therefore, we chose alchemical transformation calculations (eq S11), as their accuracy is comparable to other methods (Table S6), while they are computationally more efficient than the double decoupling method (eq S10) as fewer steps are involved. Employing the final scaling factors (Table 1, main text), we calculate Mg^{2+} binding affinities toward the DMP by transforming the Mg^{2+} ion for each water models into a reference ion (eq S11) and using forward and backward transformations. For the reference ion, we chose the parameters by Mamatkulov-Schwierz for Ca^{2+} in TIP3P water[1] since we already had well converged results based on our previous work (Table S6).

The transformation was performed in 20 iterative steps gradually switching the Lennard-Jones parameters from the respective Mg^{2+} model to the reference ion and vice versa both in solution as well as at its binding site (yielding $\Delta G^{\rm solv}_{\rm ion\to ref}$ and $\Delta G^{\rm BS}_{\rm ref\to ion}$ from eq S11).

$\Delta G^{0}_{\rm ref}$ for each water model was obtained from integration of the potentials of mean force (Figure S8) using eq S12 (simulation parameters are again given in Section S1.2 and Section S1.6) and the values are listed in Table S8.

Employing the respective reference binding affinities, we check for convergence by performing the transformation for each Mg^{2+} model corresponding to one of the five waters both in forward as well as in backward direction (Tables S9 and S10).

Both methods yielded identical results within error (Tables S8, S9, and S10). Errors were calculated from block averaging by dividing the trajectory of the alchemical transformation into 5 blocks.

S2 Supplementary results

S2.1 Transferability

Table S7: Parameters and single-ion properties after converting *microMg*(TIP3P) and *nanoMg*(TIP3P) into *microMg*(TIP3P|W) and *nanoMg*(TIP3P|W) using Lorentz-Berthelot combination rules (eq 2, main text, unscaled). $\sigma_{\rm ii}$, $\varepsilon_{\rm ii}$, $\sigma_{\rm io}$, $\varepsilon_{\rm io}$ are the ion-ion and ion-water LJ parameters single-ion properties. $\Delta G_{\rm solv}$, R_1, and n_1 are the solvation free energy of the neutral $MgCl_2$ ion-pair, the radius of the first hydration shell, and the coordination number of the first hydration shell, respectively.

	$\sigma_{\rm ii}$ [nm]	$\varepsilon_{\rm ii}$ [kJ/mol]	$\sigma_{\rm io}$ [nm]	$\varepsilon_{\rm io}$ [kJ/mol]	$\Delta G_{\rm solv}$ [kJ/mol]	R_1 [nm]	n_1
microMg(TIP3P|SPC/E)	0.1019	235.80	0.2101	13.75	-2534.9 ± 1	0.206 ± 0.004	6
nanoMg(TIP3P|SPC/E)	0.1025	389.80	0.2106	17.50	-2535.0 ± 1	0.210 ± 0.004	6
microMg(TIP3P|TIP3P-fb)	0.1019	235.80	0.2107	13.77	-2536.1 ± 1	0.208 ± 0.004	6
nanoMg(TIP3P|TIP3P-fb)	0.1025	389.80	0.2112	17.52	-2535.5 ± 1	0.211 ± 0.004	6
microMg(TIP3P|TIP4P/2005)	0.1019	235.80	0.2097	15.01	-2451.2 ± 1	0.210 ± 0.004	6
nanoMg(TIP3P|TIP4P/2005)	0.1025	389.80	0.2102	19.10	-2455.8 ± 1	0.213 ± 0.004	6
microMg(TIP3P|TIP4P-D)	0.1019	235.80	0.2101	14.49	-2474.7 ± 1	0.211 ± 0.004	6
nanoMg(TIP3P|TIP4P-D)	0.1025	389.80	0.2106	20.99	-2483.1 ± 1	0.214 ± 0.004	6
microMg(TIP3P|TIP4P-Ew)	0.1019	235.80	0.2100	14.07	-2462.9 ± 1	0.208 ± 0.004	6
nanoMg(TIP3P|TIP4P-Ew)	0.1025	389.80	0.2105	17.90	-2465.4 ± 1	0.212 ± 0.004	6
exp.					-2532 [12]	0.209 ± 0.004 [11]	6 [11]

S2.2 Isosurfaces

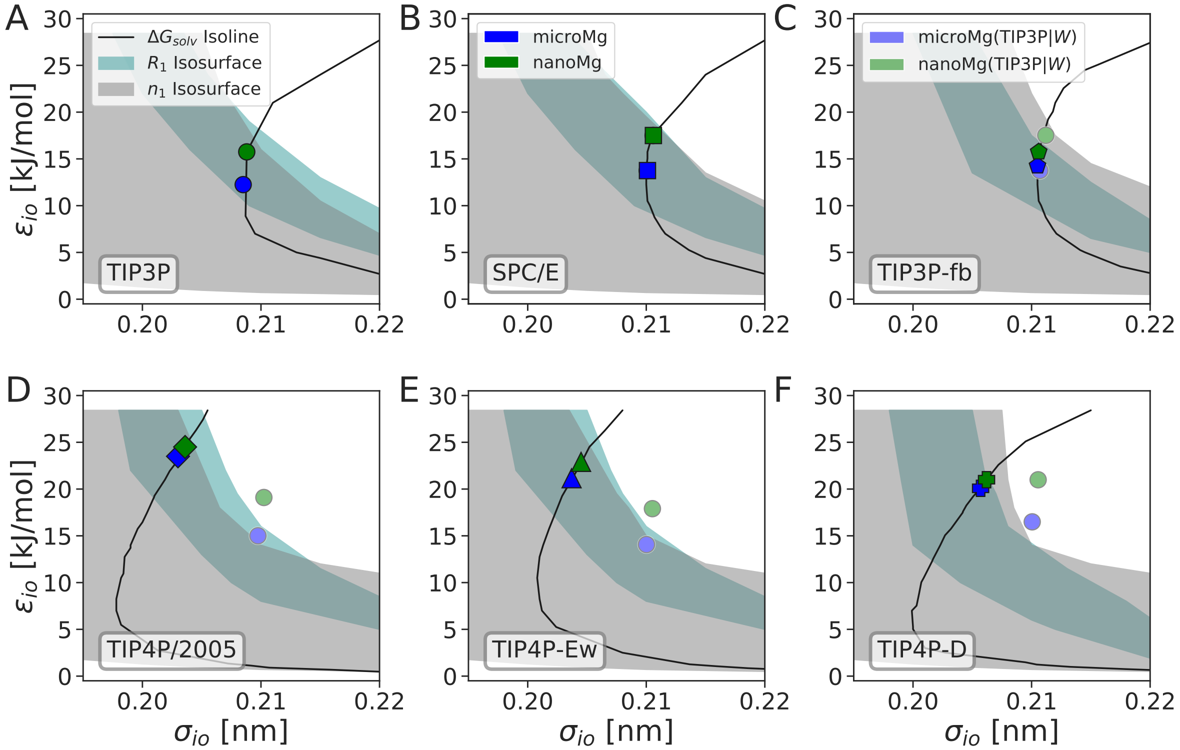

Figure S3: Solvation free energy ΔG_{solv}, radius of the first hydration shell R_1, and coordination number n_1 isosurfaces in σ_{io} - $\varepsilon_{\mathrm{io}}$ space for the interaction of the *microMg* and *nanoMg* parameters of various water models, including the force fields for (A) TIP3P [45] from the literature,[6] (B) SPC/E,[46] (C) TIP3P-fb,[25] (D) TIP4P/2005,[47] (E) TIP4P-Ew,[26] and (F) TIP4P-D.[48] Transparent circles indicate converted *microMg*(TIP3P|W) and *nanoMg*(TIP3P|W) parameters, using the ion-ion parameter sets, σ_{ii} and $\varepsilon_{\mathrm{ii}}$, of TIP3P water[6] and Lorentz-Berthelot combination rules (eq 2, main text, unscaled) to gain the effective water-ion parameter sets, σ_{io} and $\varepsilon_{\mathrm{io}}$, of the respective water model W (Table S7). In parameter regions far off the ΔG_{solv} isolines, grid points are sparser. Therefore, the R_1 and n_1 surfaces are less accurate in those regions.

S2.3 Lennard-Jones interaction potentials

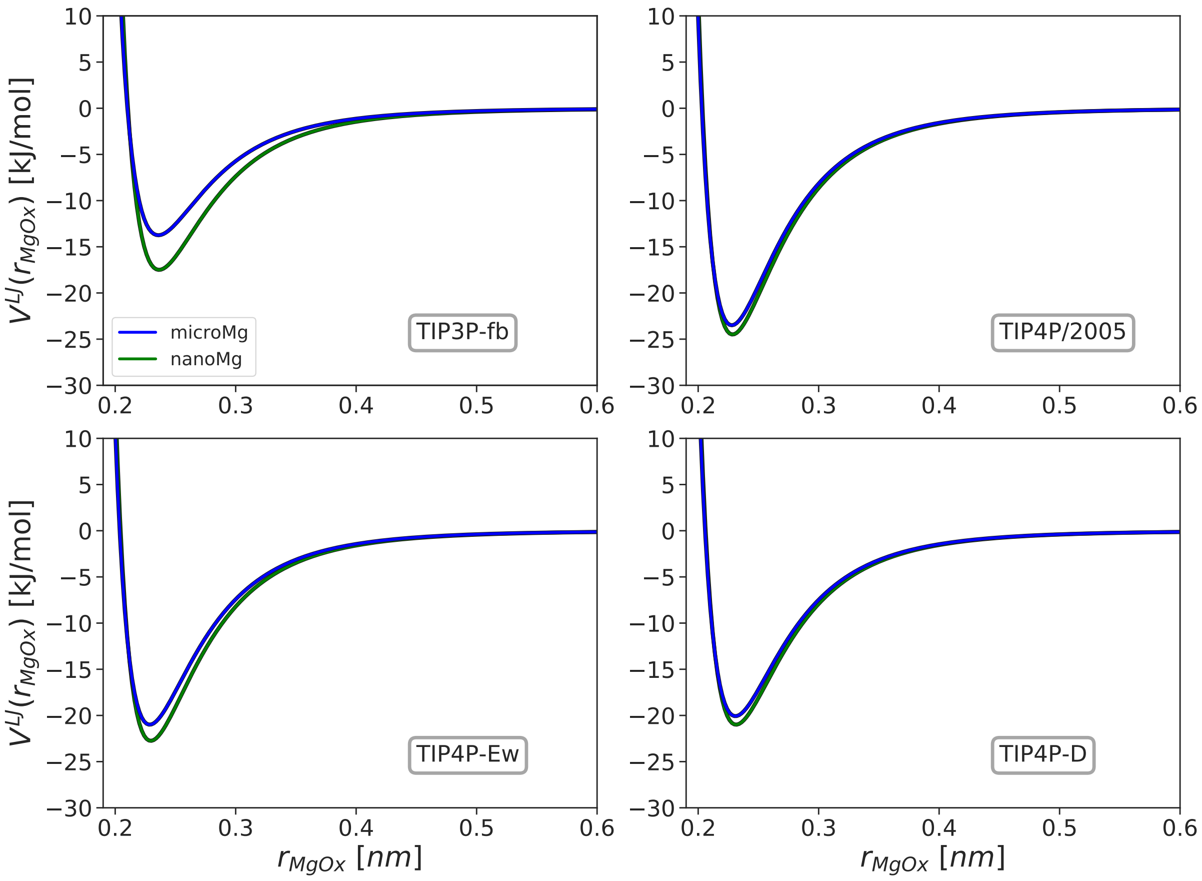

Figure S4: Lennard-Jones interaction potential V^{LJ} as function of the Mg^{2+}-oxygen distance r_{MgOw} for different Mg^{2+} force fields and (A) TIP3P-fb,[25] (B) TIP4P/2005,[47] (C) TIP4P-Ew,[26] and (D) TIP4P-D[48] water models.

S2.4 One-dimensional free energy profiles for Mg^{2+}-water interactions

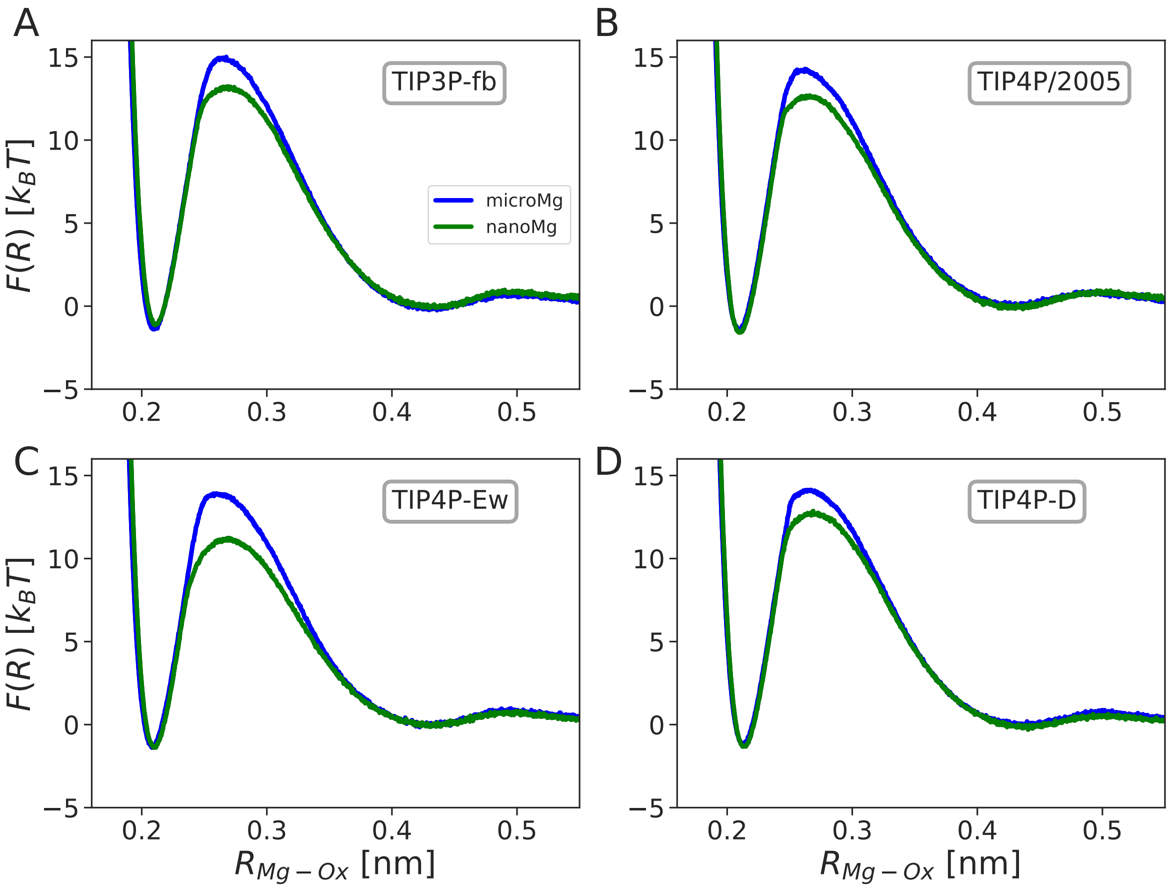

Figure S5: One-dimensional free energy profiles as a function of the distance between Mg^{2+} and the leaving water molecule $R_{\mathrm{Mg-Ox}}$ for different force fields in combination with (A) TIP3P-fb,[25] (B) TIP4P/2005,[47] (C) TIP4P-Ew,[26] and (D) TIP4P-D[48] water models.

S2.5 Two-dimensional free energy profiles for Mg^{2+}-water interactions

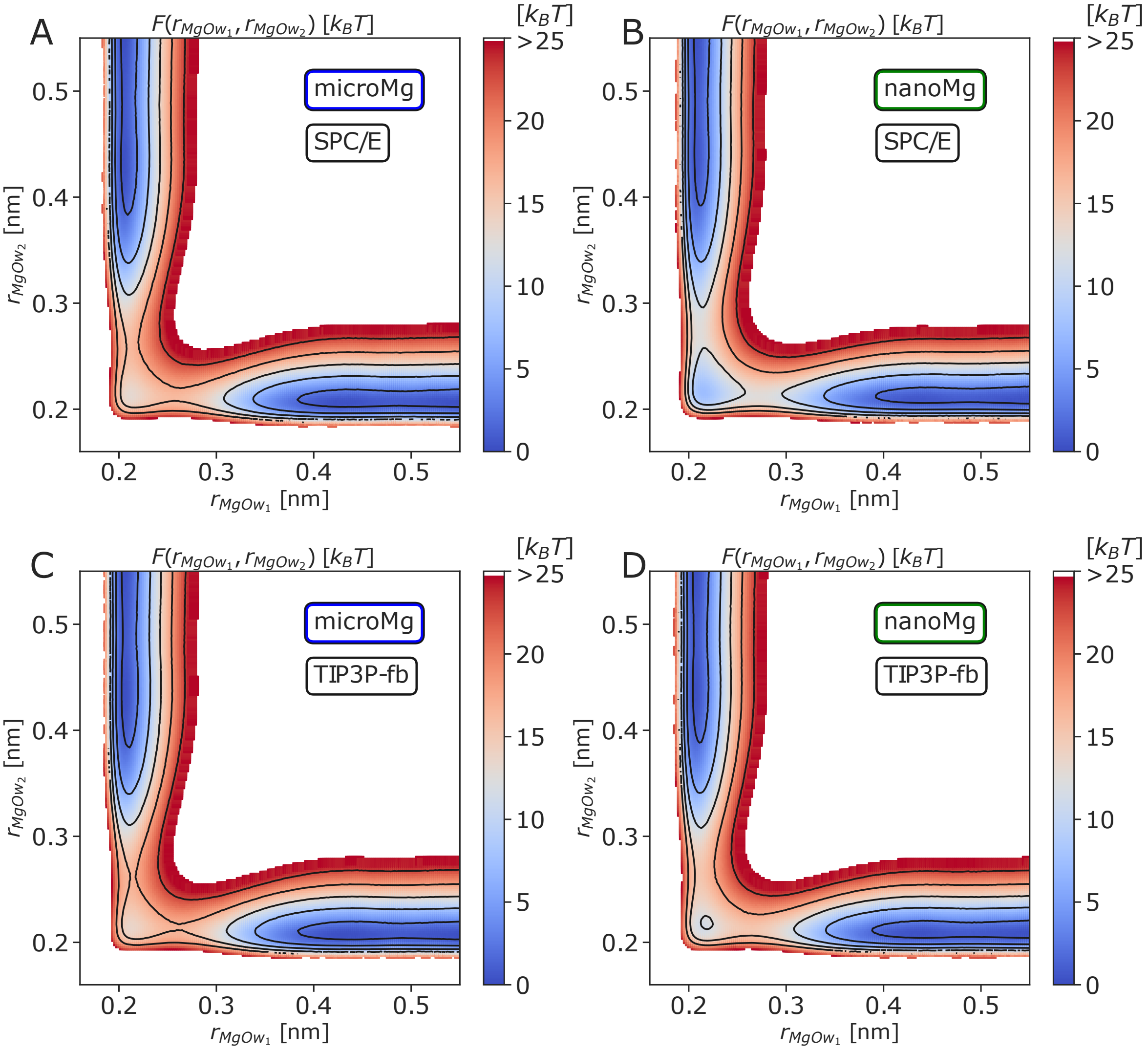

Figure S6: Two-dimensional free energy profiles as a function of the distance between Mg^{2+} and the leaving water molecule $r_{\mathrm{MgOw_1}}$ and Mg^{2+} and the entering water molecule $r_{\mathrm{MgOw_2}}$ for different force fields in combination with (A,B) SPC/E and (C,D) TIP3P-fb water models. The free energy profiles were calculated via umbrella sampling using additional restraints (see ref.[6] for more details).

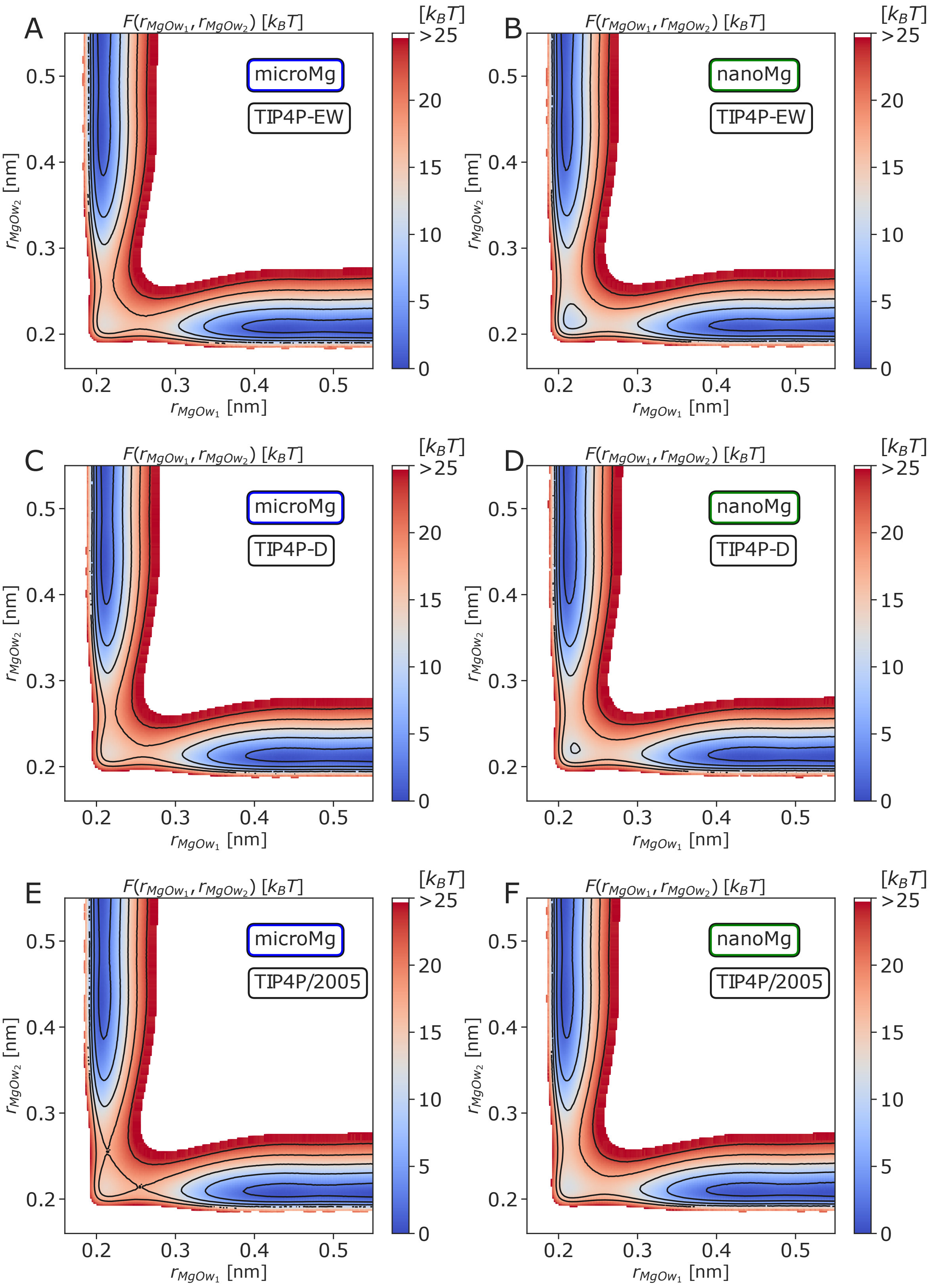

Figure S7: Two-dimensional free energy profiles as a function of the distances between Mg^{2+} and the leaving water molecule $r_{\mathrm{MgOw_1}}$ and Mg^{2+} and the entering water molecule $r_{\mathrm{MgOw_2}}$ for different force fields in combination with (A,B) TIP4P-Ew, (C,D) TIP4P-D and (E,F) TIP4P/2005 water models. The free energy profiles were calculated via umbrella sampling using additional restraints (see ref.[6] for more details).

S2.6 Binding affinities

Two strategies were proceeded to validate binding affinities values, as the calculation of binding affinities is prone to errors even for small systems, like the one we are using here. Both methods yielded nearly identical results within error (Tables 5, main text, S9, and S10).

First strategy to calculate binding affinities: Integration of the free energy profiles along the distance between the Mg^{2+} ion and the dimethylphosphate (DMP) molecule. Resulting properties are listed in Table 5 in the main text.

Second strategy to calculate binding affinities: Alchemical transformation between Mg^{2+} and an divalent reference atom of known binding affinity. As reference ion (Table S8), we choose the parameters of Ca^{2+} in TIP3P water obtained earlier.[1]

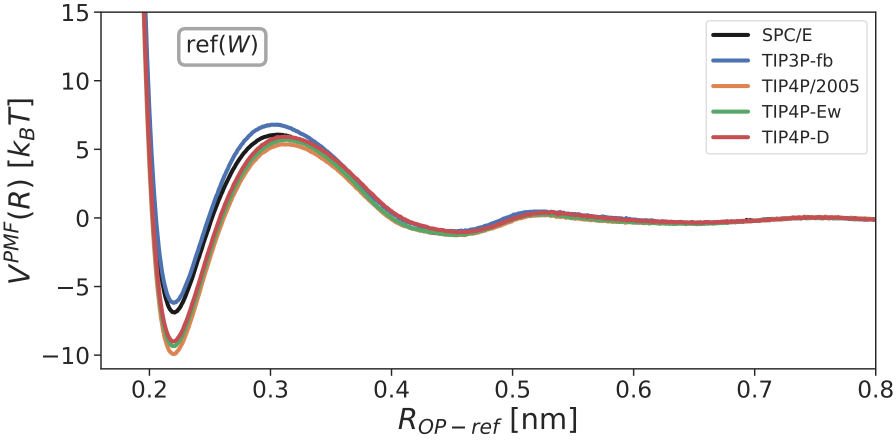

Figure S8: One-dimensional potential of mean force V^{PMF} as a function of the distance $R_{\mathrm{OP-ref}}$ between the divalent reference ion and one of the non-bridging phosphate oxygens of the DMP in different water models. The free energy profile was calculated via umbrella sampling using additional restraints (see ref.[6] for more details). Afterwards a Jacobian correction was applied (eq S8).

Table S8: Binding affinity $\Delta G^0_{\mathrm{ref}}$ obtained for the divalent reference ion (parameters for Ca^{2+} from Mamatkulov-Schwierz in TIP3P[1]) in the different water models via integration of their potentials of mean force (Figure S8).

water model	$\Delta G^0_{\mathrm{ref}}$ [$\mathrm{k_B T}$]
SPC/E	-4.561 ± 0.174
TIP3P-fb	-3.881 ± 0.132
TIP4P/2005	-7.591 ± 0.065
TIP4P-Ew	-7.057 ± 0.090
TIP4P-D	-6.703 ± 0.210

Table S9: Binding affinity ΔG_b^0 obtained from forward alchemical transformation. The values given for $\Delta G^\mathrm{solv}_{\mathrm{Mg}^{2+}\to\mathrm{ref}}$ and $\Delta G^\mathrm{bind}_{\mathrm{ref}\to\mathrm{Mg}^{2+}}$ are obtained from block averaging for 5 blocks of 3 ns long windows each. $\Delta G^\mathrm{bind}_{\mathrm{ref}\to\mathrm{Mg}^{2+}}$, $\Delta G^\mathrm{bind}_{\mathrm{Mg}^{2+}\to\mathrm{ref}}$ and hence ΔG_b^0 are shown for the final scaling factor combination $\lambda^\mathrm{RNA}_{\sigma,\varepsilon}$ (Table 2, main text). R_b is obtained from the last 15 ns window of the alchemical transformation calculation, the error here indicates the standard deviation of the distribution.

	ΔG_b^0 [$\mathrm{k_BT}$]	$\Delta G^\mathrm{solv}_{\mathrm{Mg}^{2+}\to\mathrm{ref}}$ [$\mathrm{k_BT}$]	$\Delta G^\mathrm{bind}_{\mathrm{ref}\to\mathrm{Mg}^{2+}}$ [$\mathrm{k_BT}$]	R_b [nm]
microMg(SPC/E)	-0.873 ± 0.4	131.776 ± 0.003	-128.088 ± 0.003	0.209 ± 0.004
nanoMg(SPC/E)	-1.033 ± 0.4	131.766 ± 0.003	-128.238 ± 0.003	0.208 ± 0.004
microMg(TIP3P-fb)	-0.789 ± 0.4	133.557 ± 0.006	-130.465 ± 0.003	0.208 ± 0.004
nanoMg(TIP3P-fb)	-0.976 ± 0.4	133.674 ± 0.006	-130.770 ± 0.006	0.208 ± 0.004
microMg(TIP4P/2005)	0.138 ± 0.4	161.893 ± 0.008	-154.165 ± 0.007	0.207 ± 0.004
nanoMg(TIP4P/2005)	-0.632 ± 0.4	161.396 ± 0.007	-154.437 ± 0.007	0.207 ± 0.004
microMg(TIP4P-Ew)	-0.897 ± 0.4	154.691 ± 0.007	-148.532 ± 0.007	0.208 ± 0.004
nanoMg(TIP4P-Ew)	-0.945 ± 0.4	154.497 ± 0.003	-148.384 ± 0.006	0.208 ± 0.004
microMg(TIP4P-D)	-0.677 ± 0.4	158.560 ± 0.007	-152.535 ± 0.006	0.207 ± 0.004
nanoMg(TIP4P-D)	-0.775 ± 0.4	158.550 ± 0.006	-152.622 ± 0.006	0.207 ± 0.004
exp.	-1.036 [15]	n.a.	n.a.	0.206 - 0.208 [49]

Table S10: Binding affinity ΔG_b^0 obtained from backward alchemical transformation. The values given for $\Delta G^\mathrm{solv}_{\mathrm{ref}\to\mathrm{Mg}^{2+}}$ and $\Delta G^\mathrm{bind}_{\mathrm{Mg}^{2+}\to\mathrm{ref}}$ are obtained from block averaging for 5 blocks of 3 ns long windows each. $\Delta G^\mathrm{bind}_{\mathrm{Mg}^{2+}\to\mathrm{ref}}$ and hence ΔG_b^0 are shown for the final scaling factor combination $\lambda^\mathrm{RNA}_{\sigma,\varepsilon}$ (Table 2, main text). R_b is obtained from the first 15 ns window of the alchemical transformation calculation, the error here indicates the standard deviation of the distribution.

	ΔG_b^0 [$\mathrm{k_BT}$]	$\Delta G^\mathrm{solv}_{\mathrm{ref}\to\mathrm{Mg}^{2+}}$ [$\mathrm{k_BT}$]	$\Delta G^\mathrm{bind}_{\mathrm{Mg}^{2+}\to\mathrm{ref}}$ [$\mathrm{k_BT}$]	R_b [nm]
microMg(SPC/E)	-0.987 ± 0.4	-131.606 ± 0.003	128.032 ± 0.003	0.209 ± 0.004
nanoMg(SPC/E)	-1.097 ± 0.4	-131.867 ± 0.003	128.403 ± 0.003	0.208 ± 0.004
microMg(TIP3P-fb)	-0.953 ± 0.4	-133.431 ± 0.006	130.503 ± 0.003	0.208 ± 0.004
nanoMg(TIP3P-fb)	-0.821 ± 0.4	-133.687 ± 0.006	130.627 ± 0.003	0.209 ± 0.004
microMg(TIP4P/2005)	-0.435 ± 0.4	-161.357 ± 0.008	154.200 ± 0.007	0.207 ± 0.004
nanoMg(TIP4P/2005)	-0.974 ± 0.4	-161.162 ± 0.007	154.544 ± 0.007	0.207 ± 0.004
microMg(TIP4P-Ew)	-0.770 ± 0.4	-154.764 ± 0.003	148.478 ± 0.003	0.208 ± 0.004
nanoMg(TIP4P-Ew)	-0.589 ± 0.4	-154.582 ± 0.003	148.114 ± 0.006	0.208 ± 0.004
microMg(TIP4P-D)	-0.444 ± 0.4	-158.512 ± 0.007	152.254 ± 0.006	0.207 ± 0.004
nanoMg(TIP4P-D)	-0.687 ± 0.4	-158.678 ± 0.006	152.662 ± 0.006	0.207 ± 0.004
exp.	-1.036 [15]	n.a.	n.a.	0.206 - 0.208 [49]

S3 Bibliography

(1) Mamatkulov, S.; Schwierz, N. Force fields for monovalent and divalent metal cations in TIP3P water based on thermodynamic and kinetic properties. *J. Chem. Phys.* **2018**, *148*, 74504.

(2) Schwierz, N. Kinetic pathways of water exchange in the first hydration shell of magnesium. *J. Chem. Phys.* **2020**, *152*, 224106.

(3) Cruz-León, S.; Grotz, K. K.; Schwierz, N. Extended magnesium and calcium force field parameters for accurate ion-nucleic acid interactions in biomolecular simulations. *J. Chem. Phys.* **2021**, *154*, 171102.

(4) Nilsson, L.; Villa, A. Magnesium Ion Water Coordination and Exchange in Biomolecular Simulations. **2012**,

(5) Panteva, M. T.; Giambasu, G. M.; York, D. M. Comparison of structural, thermodynamic, kinetic and mass transport properties of Mg^{2+} ion models commonly used in biomolecular simulations. *J. Comput. Chem.* **2015**, *36*, 970–982.

(6) Grotz, K. K.; Cruz-León, S.; Schwierz, N. Optimized Magnesium Force Field Parameters for Biomolecular Simulations with Accurate Solvation, Ion-Binding, and Water-Exchange Properties. *J. Chem. Theory Comput.* **2021**, *17*, 2530–2540.

(7) Li, P.; Roberts, B. P.; Chakravorty, D. K.; Merz, K. M. Rational Design of Particle Mesh Ewald Compatible Lennard-Jones Parameters for +2 Metal Cations in Explicit Solvent. *J. Chem. Theory Comput.* **2013**, *9*, 2733–2748.

(8) Li, P.; Merz, K. M. Taking into account the ion-induced dipole interaction in the nonbonded model of ions. *J. Chem. Theory Comput.* **2014**, *10*, 289–297.

(9) Mamatkulov, S.; Fyta, M.; Netz, R. R. Force fields for divalent cations based on single-ion and ion-pair properties. *J. Chem. Phys.* **2013**, *138*, 24505.

(10) Åqvist, J. Ion-water interaction potentials derived from free energy perturbation simulations. *J. Phys. Chem.* **1990**, *94*, 8021–8024.

(11) Marcus, Y. Ionic Radii in Aqueous solutions. *Chem. Rev.* **1988**, *88*, 1475–1498.

(12) Marcus, Y. *Ion Properties*; Marcel Dekker, Inc.: New York, Basel, 1997.

(13) Neely, J.; Connick, R. Rate of Water Exchange from Hydrated Magnesium Ion. *J. Am. Chem. Soc.* **1970**, *92*, 3476–3478.

(14) Bleuzen, A.; Pittet, P.-A.; Helm, L.; Merbach, A. E. Water exchange on magnesium(II) in aqueous solution: a variable temperature and pressure ^{17}O NMR study. *Magn. Reson. Chem.* **1997**, *35*, 765–773.

(15) Sigel, R. K.; Sigel, H. A stability concept for metal ion coordination to single-stranded nucleic acids and affinities of individual sites. *Acc. Chem. Res.* **2010**, *43*, 974–984.

(16) Darden, T.; Pearlman, D.; Pedersen, L. G. Ionic charging free energies: Spherical versus periodic boundary conditions. *J. Chem. Phys.* **1998**, *109*, 10921–10935.

(17) Ryckaert, J. P.; Ciccotti, G.; Berendsen, H. J. Numerical integration of the cartesian equations of motion of a system with constraints: molecular dynamics of n-alkanes. *J. Comp. Phys.* **1977**, *23*, 327–341.

(18) Hess, B.; Bekker, H.; Berendsen, H. J. C.; Fraaije, J. G. E. M. LINCS: a linear constraint solver for molecular simulations. *J. Comput. Chem.* **1997**, *18*, 1463–1472.

(19) Berendsen, H. J.; Postma, J. P.; Van Gunsteren, W. F.; Dinola, A.; Haak, J. R. Molecular dynamics with coupling to an external bath. *J. Chem. Phys.* **1984**, *81*, 3684–3690.

(20) Bussi, G.; Donadio, D.; Parrinello, M. Canonical sampling through velocity rescaling. *J. of Chem. Phys.* **2007**, *126*.

(21) Parrinello, M.; Rahman, A. Polymorphic transitions in single crystals: A new molecular dynamics method. *J. Appl. Phys.* **1981**, *52*, 7182–7190.

(22) Yeh, I.-C.; Hummer, G. System-Size Dependence of Diffusion Coefficients and Viscosities from Molecular Dynamics Simulations with Periodic Boundary Conditions. *J. Phys. Chem. B* **2004**, *108*, 15873–15879.

(23) Vega, C.; Abascal, J. L. Simulating water with rigid non-polarizable models: A general perspective. *Phys. Chem. Chem. Phys.* **2011**, *13*, 19663–19688.

(24) von Bülow, S.; Siggel, M.; Linke, M.; Hummer, G. Dynamic cluster formation determines viscosity and diffusion in dense protein solutions. *Proc. Natl. Acad. Sci.* **2019**, *116*, 9843–9852.

(25) Wang, L. P.; Martinez, T. J.; Pande, V. S. Building force fields: An automatic, systematic, and reproducible approach. *J. Phys. Chem. Lett.* **2014**, *5*, 1885–1891.

(26) Horn, H. W.; Swope, W. C.; Pitera, J. W.; Madura, J. D.; Dick, T. J.; Hura, G. L.; Head-Gordon, T. Development of an improved four-site water model for biomolecular simulations: TIP4P-Ew. *J. Chem. Phys.* **2004**, *120*, 9665–9678.

(27) Wynne-Jones, W. F. K.; Eyring, H. The Absolute Rate of Reactions in Condensed Phases. *J. Chem. Phys.* **1935**, *3*, 492–502.

(28) Wigner, E. The Transition State Method. *Trans. Faraday Soc.* **1937**, 29–41.

(29) Frenkel, D.; Smit, B. *Understanding Molecular Simulation: From Algorithms to Applications*, 2nd ed.; Elsevier Science, 2001.

(30) Weerasinghe, S.; Smith, P. E. A Kirkwood-Buff derived force field for sodium chloride in water. *J. Chem. Phys.* **2003**, *119*, 11342–11349.

(31) Fyta, M.; Netz, R. R. Ionic force field optimization based on single-ion and ion-pair solvation properties: Going beyond standard mixing rules. *J. Chem. Phys.* **2012**, *136*.

(32) Kirkwood, J. G.; Buff, F. P. The Statistical Mechanical Theory of Solutions. I. *J. Chem. Phys.* **1951**, *19*, 774–777.

(33) Lee Warren, G.; Patel, S. Hydration free energies of monovalent ions in transferable intermolecular potential four point fluctuating charge water: An assessment of simulation methodology and force field performance and transferability. *J. Chem. Phys.* **2007**, *127*.

(34) Torrie, G. M.; Valleau, J. P. Monte Carlo free energy estimates using non-Boltzmann sampling: Application to the sub-critical Lennard-Jones fluid. *Chem. Phys. Lett.* **1974**, *28*, 578–581.

(35) and Torrie, G.; Valleau, J. Nonphysical sampling distributions in Monte Carlo free-energy estimation: Umbrella sampling. *J. Comput. Phys.* **1977**, *23*, 187.

(36) Kumar, S.; Rosenberg, J. M.; Bouzida, D.; Swendsen, R. H.; Kollman, P. A. The weighted histogram analysis method for free-energy calculations on biomolecules. I. The method. *J. Comput. Chem.* **1992**, *13*, 1011–1021.

(37) Hess, B. P-LINCS: A Parallel Linear Constraint Solver for Molecular Simulation. *J. Chem. Theory Comput.* **2008**, *4*, 116–122.

(38) Case, D. A.; Belfon, K.; Ben-Shalom, I. Y.; Brozell, S. R.; Cerutti, D. S.; Cheatham, T. E. I.; Cruzeiro, V. W. D.; Darden, T.; Duke, R. E.; Giambasu, G.; Gilson, M. K.; Gohlke, H.; Goetz, A. W.; Harris, R.; Izadi, P. A.; Izmailov, S.; Kasavajhala, K.; Kovalenko, A.; Krasny, R.; Kurtzman, T.; Lee, T. S.; LeGrand, S.; Li, P.; Lin, C.; Liu, J.; Luchko, T.; Luo, R.; Man, V.; Merz, K. M.; Miao, Y.; Mikhailovskii, O.; Monard, G.; Nguyen, H.; Onufriev, A.; Pan, F.; Pantano, S.; Qi, R.; Roe, D. R.; Roitberg, A.; Sagui, C.; Schott-Verdugo, S.; Shen, J.; Simmerling, C. L.; Skrynnikov, N. R.; Smith, J.; Swails, J.; Walker, R. C.; Wang, J.; Wilson, L.; Wolf, R. M.; Wu, X.; Xiong, Y.; Xue, Y.; York, D. M.; Kollman, P. A. Amber 2018. 2018; `https://ambermd.org/AmberTools.php`.

(39) Tribello, G. A.; Bonomi, M.; Branduardi, D.; Camilloni, C.; Bussi, G. Plumed 2: New feathers for an old bird. *Comput. Phys. Commun.* **2014**, *185*, 604–613.

(40) Hess, B.; van der Vegt, N. F. A. Cation specific binding with protein surface charges. *Proc. Natl. Acad. Sci.* **2009**, *106*, 13296–13300.

(41) Cruz-León, S.; Schwierz, N. Hofmeister Series for Metal-Cation-RNA Interactions: The Interplay of Binding Affinity and Exchange Kinetics. *Langmuir* **2020**, *36*, 5979–5989.

(42) Gilson, M. K.; Given, J. A.; Bush, B. L.; McCammon, J. A. The statistical-thermodynamic basis for computation of binding affinities: a critical review. *Biophys. J.* **1997**, *72*, 1047–1069.

(43) Chipot, C. *Free energy calculations*; Springer Science Business Media: Heidelberg, Berlin, 2007.

(44) Cruz-León, S.; Vanderlinden, W.; Müller, P.; Forster, T.; Staudt, G.; Lin, Y.; Lipfert, J.; Schwierz, N. Twisting DNA by Salt. *bioRxiv2021.07.14.452306* **2021**, 21–26.

(45) Jorgensen, W. L.; Chandrasekhar, J.; Madura, J. D.; Impey, R. W.; Klein, M. L. Comparison of simple potential functions for simulating liquid water. *J. Chem. Phys.* **1983**, *79*, 926–935.

(46) Berendsen, H. J.; Grigera, J. R.; Straatsma, T. P. The missing term in effective pair potentials. *J. Phys. Chem.* **1987**, *91*, 6269–6271.

(47) Abascal, J. L.; Vega, C. A general purpose model for the condensed phases of water: TIP4P/2005. *J. Chem. Phys.* **2005**, *123*.

(48) Piana, S.; Donchev, A. G.; Robustelli, P.; Shaw, D. E. Water dispersion interactions strongly influence simulated structural properties of disordered protein states. *J. Phys. Chem. B* **2015**, *119*, 5113–5123.

(49) Leonarski, F.; D'Ascenzo, L.; Auffinger, P. Mg^{2+} ions: Do they bind to nucleobase nitrogens? *Nucleic Acids Res.* **2017**, *45*, 987–1004.
